# The MYO1F interactome reveals ASAP1, CD2AP and SH3KBP1 as novel adaptor proteins in podosomes and phagosomes

**DOI:** 10.1242/jcs.264357

**Published:** 2025-12-22

**Authors:** Susan D. Arden, Eva Pennink, András Lakatos, Gillian M. Griffiths, Anna H. Lippert, Folma Buss

**Affiliations:** ^1^ Cambridge Institute for Medical Research, Department of Clinical Biochemistry, University of Cambridge, Cambridge Biomedical Campus, The Keith Peters Building, Hills Road, Cambridge CB2 0XY, UK; ^2^ John van Geest Centre for Brain Repair, Department of Clinical Neurosciences, University of Cambridge, Cambridge CB2 0PY, UK; ^3^ MRC-WT Cambridge Stem Cell Institute, Cambridge Biomedical Campus, Cambridge CB2 0AW, UK; ^4^ Institute for Systems Immunology, Julius-Maximilians University Würzburg, 97078 Würzburg, Germany

**Keywords:** Myosin, Actin, Podosomes, Phagocytosis, Microglia

## Abstract

MYO1F, a long-tailed myosin of class I, is selectively expressed in immune cells and upregulated in microglia associated with neurodegenerative pathogenesis. Myosin motor functions are regulated by adaptor proteins that mediate cargo attachment and motor recruitment. To define the MYO1F interactome, we used *in situ* proximity labelling and proteomics in human myeloid cells. We identified a distinct SH3-domain-dependent adaptor module comprising CD2AP, ASAP1, SH3BP2 and SH3KBP1 (herein termed the CASS group of proteins). Interestingly, CD2AP is an Alzheimer's disease (AD) risk gene upregulated in the microglia of individuals with AD, which are implicated in phagocytic responses to amyloid-β. Structural modelling and mutagenesis confirmed multivalent proline-rich motif interactions between the CASS group of proteins and the MYO1F SH3 domain. Additional binding partners associate with the MYO1F pleckstrin homology (PH) domain. Immunofluorescence revealed colocalisation of MYO1F and the CASS group of proteins at actin-rich podosomes and phagocytic cups in macrophages and microglia. Functional assays demonstrated that MYO1F recruitment to the phagocytic cup requires motor activity and intact PH and SH3 domains. We provide the first MYO1F interactome identifying adaptor proteins for MYO1F in podosomes and during phagocytosis, offering new insights into its function in disease-associated microglia during neurodegeneration.

## Teaser

**Highlighted Article:** The MYO1F interactome identifies ASAP1, CD2AP and SH3BP2 as novel adaptor proteins in podosomes and during phagocytosis, offering new insights into MYO1F function in neuroinflammatory microglia.

## INTRODUCTION

Within eukaryotic cells, myosin motor proteins move cargo over short distances along actin tracks, regulate plasma membrane dynamics through the actin cortex and provide flexible tethering of organelles, vesicles and protein complexes to the actin cytoskeleton (Fili and Toseland, 2019; Hartman and Spudich, 2012; Masters et al., 2017). Myosins of class I are widely expressed monomeric single-headed motors (McIntosh and Ostap, 2016). The biological functions of class I myosins are at the membrane–cytoskeleton interface, where they provide mechanical force and tension that allows remodelling of membranes relative to the underlying actin network. They play important roles in plasma membrane dynamics and membrane trafficking as well as in organisation of the actin cytoskeleton (Girón-Pérez et al., 2019; Greenberg and Ostap, 2013).

In humans, a total of eight class I myosins are expressed (MYO1A–MYO1H), which are subdivided into the short-tailed and the long-tailed myosins (Foth et al., 2006). All myosins of class I can directly interact with cell membranes through the pleckstrin homology domain (PH) present within the tail homology 1 (TH1) domain (McConnell and Tyska, 2010). Two additional domains are specific to the long-tailed MYO1E and MYO1F, a proline-rich tail homology 2 (TH2) domain and a C-terminal Src homology 3 (SH3) domain (Navines-Ferrer and Martin, 2020; Stöffler et al., 1995). MYO1E can be found in a wide variety of cell types and tissues with enriched expression in the kidney and intestine (Liu et al., 2024; Stöffler et al., 1995; www.proteinatlas.org), whereas MYO1F expression is restricted to blood and immune cells, including peripheral lymphoid and myeloid cells, but also brain-resident microglia (Kim et al., 2006; Matarin et al., 2015; Salvermoser et al., 2018; Zhang et al., 2013).

Microglia are the primary immune cells of the central nervous system, playing a crucial role in the pathophysiology of various neurodegenerative diseases (Gao et al., 2023). Both in humans with Alzheimer's disease (AD) and murine models of AD and frontotemporal dementia/amyotrophic lateral sclerosis (FTD/ALS), MYO1F is significantly upregulated in microglia compared to healthy controls. This alteration in MYO1F expression parallels changes observed in established AD- and FTD/ALS-associated risk genes, such as TREM2 and GRN (Matarin et al., 2015; Zhang et al., 2020). Furthermore, whole-genome gene expression profiling has identified MYO1F as a key node within immune-related networks in individuals with late-onset AD (Zhang et al., 2013). Additionally, systems biology analysis suggests that MYO1F is one of the seven microglia-specific genes potentially involved in a shared molecular pathway underlying the human ageing process and multiple neurodegenerative diseases such as AD, Parkinson's disease and Huntington's disease (Mukherjee et al., 2019).

In peripheral myeloid cells, MYO1F deficiency results in increased adhesion and reduced motility of neutrophils *in vitro* (Kim et al., 2006) alongside a significant reduction in extravasation, attributed to the incapacity of neutrophils to deform their nucleus while squeezing through endothelial cell barriers (Salvermoser et al., 2018). Another study shows that MYO1F mediates intercellular adhesion, which allows intestinal macrophage differentiation to a M1 phenotype. Importantly, in a mouse model of colitis, the absence of MYO1F causes a reduction in proinflammatory cytokine secretion, less tissue damage and increased tissue repair (Piedra-Quintero et al., 2019).

MYO1E and MYO1F are present in podosomes (Cervero et al., 2012) and invadopodia (Ouderkirk and Krendel, 2014), which are micrometre-sized, actin-rich structures critical for cell adhesion, matrix degradation and mechanosensing (Alonso et al., 2019; Linder et al., 2023). While invadosomes are a feature of invasive cells (Boateng and Huttenlocher, 2012), podosomes are predominantly found in monocytic cells (Linder et al., 1999) but are also observed in smooth muscle (Burgstaller and Gimona, 2004) and endothelial cells (Moreau et al., 2003). MYO1E has been shown to negatively regulate actin polymerisation at the ventral layer of podosomes, and both MYO1E and MYO1F control podosome dynamics and promote macrophage migration (Cervero et al., 2025; Zhang et al., 2019). Interestingly, actin-rich podosome-like structures have also been identified at the leading edge of the phagocytic cup (Herron et al., 2022; Linder and Barcelona, 2023). MYO1E and MYO1F contained within these phagocytic podosomes are thought to tether the plasma membrane to the underlying actin cortex, thereby increasing membrane tension (Barger et al., 2022; Vorselen et al., 2021). The absence of MYO1F and/or MYO1E results in increased actin polymerisation, leading to densely packed actin filaments that consequently slow closure of the phagocytic cup (Barger et al., 2019).

In this study, we employ *in situ* proximity labelling using BioID to characterise the overall interactome of both MYO1F and MYO1E and to identify adaptor proteins that facilitate the biological functions of these myosins in podosomes and during phagocytosis. Our analysis identifies binding partners common to both myosins across different cell types, highlighting functional overlap between these two motors, as well as a number of protein complexes unique to either MYO1F or MYO1E. We determined the essential MYO1F binding domains for different adaptor proteins, using a series of MYO1F mutants either lacking the SH3 domain (ΔSH3) or with point mutations in the PH domain (PHmut) (Patino-Lopez et al., 2010), which abolishes membrane binding. We verified several novel MYO1F binding partners, such as SH3 domain-containing kinase-binding protein 1 (SH3KBP1) (Dikic, 2002; Take et al., 2000), CD2-associated protein (CD2AP) (Dikic, 2002; Kirsch et al., 1999) and ArfGAP with SH3 domain, ankyrin repeat and PH domain 1 (ASAP1) (Brown et al., 1998; Luo et al., 2019). ASAP1 has been shown to regulate podosome assembly (Bharti et al., 2007; Shiba and Randazzo, 2011), and here we show that it colocalises with MYO1F in ventral podosomes in macrophages and human microglia. The AD risk gene CD2AP and its close homologue SH3KBP1 colocalise with MYO1F in the phagocytic cup during phagocytosis. The MYO1F interactome also includes the previously identified binding partner SH3 domain-binding protein 2 (SH3BP2) (Deckert and Rottapel, 2006; Navines-Ferrer et al., 2019; Ueki et al., 2001), validating our experimental approach. This work underscores the diverse interaction network of MYO1F, providing new insights into the molecular mechanisms that enable this multifunctional motor protein to participate in a number of cellular processes. Notably, our findings suggest potential roles for MYO1F in neurodegeneration, as variants of two of its adaptor proteins, CD2AP and SH3KBP1, have been implicated in late-onset AD.

## RESULTS

### Identification of the MYO1F interactome using *in situ* proximity labelling

**Fig. 1.**
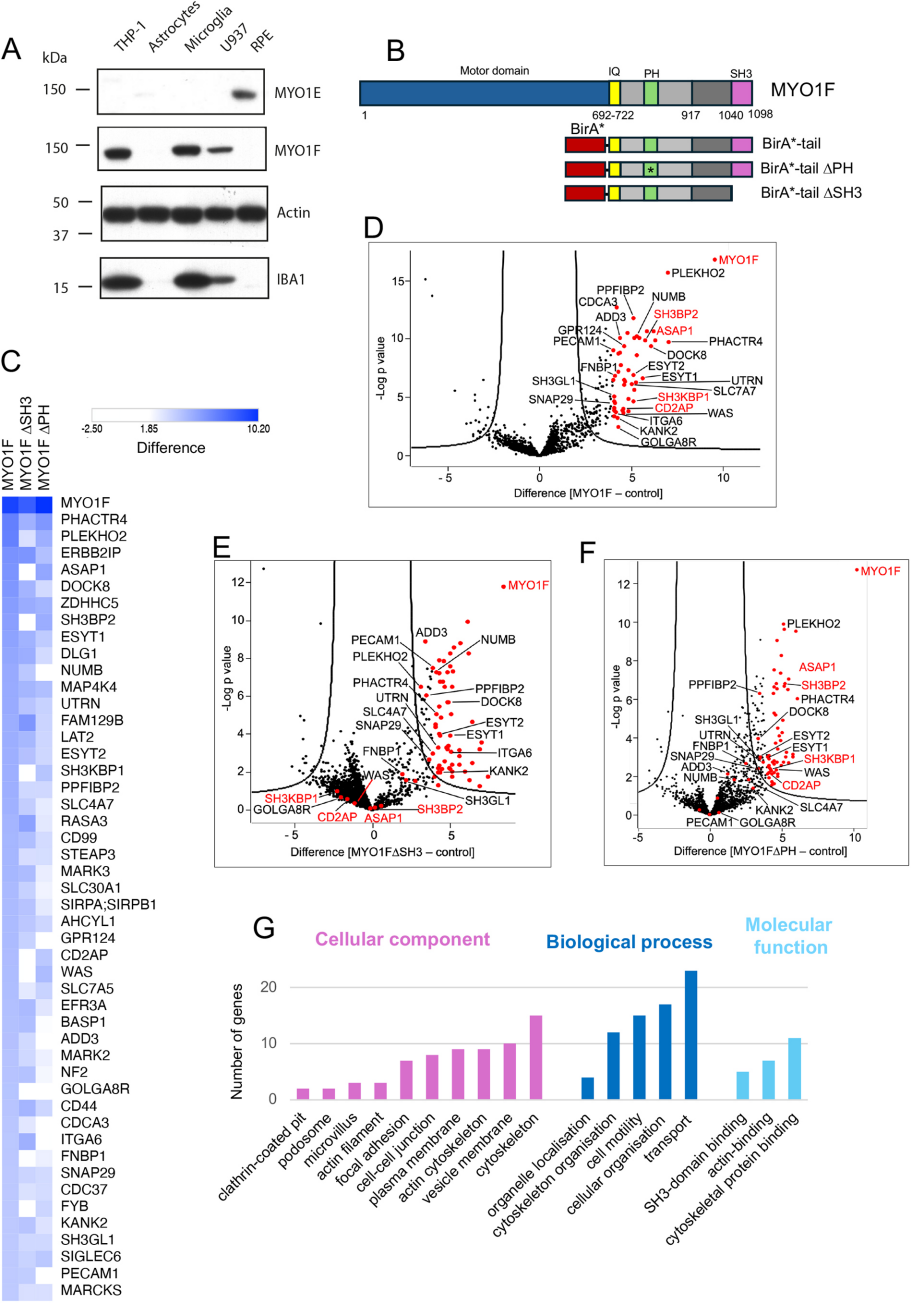
***In situ* proximity labelling reveals new binding partners for MYO1F.** (A) Expression of MYO1E and MYO1F in the THP-1, U937 and RPE cell lines, as well as primary mouse astrocytes and microglia, shown by western blotting with actin as a loading control. IBA-1 was used to identify cells of monocytic lineage such as THP-1, U937 and microglia. Blots shown are representative of three experiments. (B) Schematic diagram of MYO1F alongside the BirA*–MYO1F tail domain and mutant constructs PHmut (ΔPH) or ΔSH3 domain. IQ, calmodulin-binding motif. (C) Heat map of abundance of protein interactions with MYO1F tail domain, MYO1F tail ΔSH3 or MYO1F tail PHmut determined by BioID from U937 stable cell lines. The heatmap was generated using Morpheus software (https://software.broadinstitute.org/morpheus). Shown are protein interactions with 4-fold or greater enrichment in the BirA*–MYO1F tail experiments over the BirA*-only control. Data shown are from six independent experiments for MYO1F and three independent experiments for each of the two mutants. (D) Volcano plot of LFQ data from BirA*–MYO1F tail over BirA* control from U937 stable cell lines. The negative logarithmic *P*-value was plotted against the *t*-test difference (*n*=6). The hyperbolic cutoff curve delimitates significantly enriched proteins from common hits [FDR of 0.01 and an artificial within groups variance of 2]. Proteins of specific interest are labelled in red. The complete list is given in Table S1. (E) Volcano plot of LFQ data from BirA*–MYO1F tail ΔSH3 over BirA* control. The negative logarithmic *P*-value was plotted against the *t*-test difference (*n*=3). The hyperbolic cutoff curve delimitates significantly enriched proteins from common hits (FDR of 0.01 and SO of 2). (F) Volcano plot of LFQ data from BirA*–MYO1F tail PHmut over BirA* control. The negative logarithmic *P*-value was plotted against the *t*-test difference (*n*=3). The hyperbolic cutoff curve delimitates significantly enriched proteins from common hits (FDR of 0.01 and SO of 2). In D, E and F, significantly enriched and other notable proteins are marked by red dots and labelled by name. (G) GO classification of the MYO1F interactome identified by *in situ* proximity labelling. GO cellular component, biological process and molecular function enrichment analyses were performed using ShinyGO software (Ge et al., 2020). A term with *P*<0.05 was considered to be significantly overrepresented. The graph shows the number of proteins in the MYO1F interactome annotated with each significantly overrepresented term.

To shed further light on the various proposed functions of MYO1F in myeloid cells and to capture potential MYO1F binding partners involved in these processes, we applied *in situ* proximity labelling to living cells under steady-state conditions. MYO1F is specifically expressed in myeloid cell lines such as THP-1 and U937 as well as in primary mouse and human induced pluripotent stem cell (iPSC)-derived microglia, whereas MYO1E is expressed in a wider variety of cell types and tissues including retinal epithelial (RPE) cells (Fig. 1A). For our *in situ* proximity labelling experiments, we generated U937 cell lines stably expressing the human MYO1F tail domain (amino acids 692–1098), which includes the neck domain with the IQ motif, the PH domain in TH1 and the C-terminal SH3 domain, tagged at the N terminus with the promiscuous biotin ligase, BirA R118G (BirA*) (Fig. 1B). We verified expression of the BirA*–MYO1F tail by immunoblotting (Fig. S1A) and showed localisation of the BirA*–MYO1F tail, as well as the BirA*–MYO1F tail PHmut, at the plasma membrane in U937 cells by immunofluorescence (Fig. S2). After labelling with biotin for 24 h, biotinylated proteins were collected by streptavidin pulldown, and enriched proteins were identified by mass spectrometry. Results from six independent experiments for BirA*–MYO1F tail and three each for both mutants, BirA*–MYO1F tail PHmut and BirA*–MYO1F tail ΔSH3, were analysed against six BirA*-only U937 control pulldowns using label-free quantification (LFQ) intensities (Fig. 1). Using a threshold of 4-fold or greater enrichment in the BirA*–MYO1F tail experiments over the BirA*-only control, we identified 47 significantly enriched proximal proteins, which are shown in Fig. 1C as a heat map of the relative abundance of each protein interaction with MYO1F. These include the only known binding partner of MYO1F, SH3BP2 (Navines-Ferrer et al., 2019), and more than 40 novel MYO1F-associated proteins, which might bind directly or as part of larger MYO1F-asociated protein complexes (Fig. 1C,D; see also Table S1).

To dissect the domain-specific interaction profiles of MYO1F, we compared the interactomes of the full-length MYO1F tail with those of its SH3- and PH-domain mutants (Fig. 1C–F). This analysis revealed a core group of adaptor proteins whose interaction with MYO1F was dependent on the SH3 domain (Fig. 1C,E). These include SH3BP2 (also called 3BP2), SH3KBP1 (also called CIN85), CD2AP (also called CMS) and ASAP1. These proteins, characterised by proline-rich regions or SH3 domains themselves, form a distinct protein module we termed the CASS group of proteins (CD2AP, ASAP1, SH3BP2, SH3KBP1). The MYO1F interactome contains three further proteins that bind to the SH3 domain: the Wiskott–Aldrich syndrome protein (WAS, also known as WASP) (Derry et al., 1994; Lee et al., 2017), formin-binding protein 1 (FNBP1) (Aspenström, 2010; Katoh and Katoh, 2004) and FYN-binding protein (FYB, also known as FYB1) (Coppolino et al., 2001; Wu et al., 2017) (Fig. 1C,E). These proteins share common pathways and functions related to the actin cytoskeleton and immune cell signalling, with FYB and WAS having been identified as part of a molecular complex during phagocytosis in macrophages (Coppolino et al., 2001). Interestingly, mutation of the PH domain in the TH1 region did not affect binding of the CASS group of proteins, but reduced binding to proteins with membrane-associating functions, such as utrophin (UTRN) (Takemitsu et al., 1991; Winder et al., 1995), a linker protein that anchors the actin cytoskeleton to the plasma membrane; ADD3 (adducin gamma) (Citterio et al., 1999), a cytoskeletal protein that promotes spectrin–actin assembly at the plasma membrane; ITGA6 (integrin alpha-6) (Georges-Labouesse et al., 1998); and PECAM1 (Feng et al., 2016; Newman et al., 1990), an endothelial cell adhesion molecule (Fig. 1C,F). These interactions likely depend on proper membrane localisation of MYO1F through its PH domain and might represent a second class of functional proteins involved in plasma membrane anchoring and tension regulation.

To better understand the biological processes, molecular functions, and cellular localisations associated with the MYO1F interactome, we performed a Gene Ontology (GO) analysis on the 47 significantly enriched binding partners that were identified in our *in situ* labelling experiment (Fig. 1G). The results show a significant enrichment for GO terms associated with cytoskeletal organisation and membrane–cytoskeleton coupling, as well as cellular component terms including vesicle membranes, focal adhesions and podosomes, reinforcing the proposed role of MYO1F at podosomes and during phagocytosis.

### Identification of the MYO1E interactome using *in situ* proximity labelling

To assess the functional interaction landscape of MYO1E in epithelial cells, we performed *in situ* proximity labelling using BirA*-fused MYO1E tail constructs in RPE cells. The human MYO1E tail (amino acids 692–1108) includes the neck domain with the IQ motif, the PH domain in TH1, the proline-rich TH2 region and the SH3 domain. RPE cell lines stably expressing the BirA*–MYO1E tail, the ΔSH3 or the PHmut constructs, were generated and validated for expression by immunoblotting (Fig. S1B) and immunofluorescence (Fig. S3), which showed that mutating the PH domain does not change localisation of this myosin at the plasma membrane. Biotinylated proteins were enriched via streptavidin pulldown following overnight biotin labelling and analysed by mass spectrometry. Data from three independent experiments for each construct were compared against six BirA*-only control pulldowns.

**Fig. 2.**
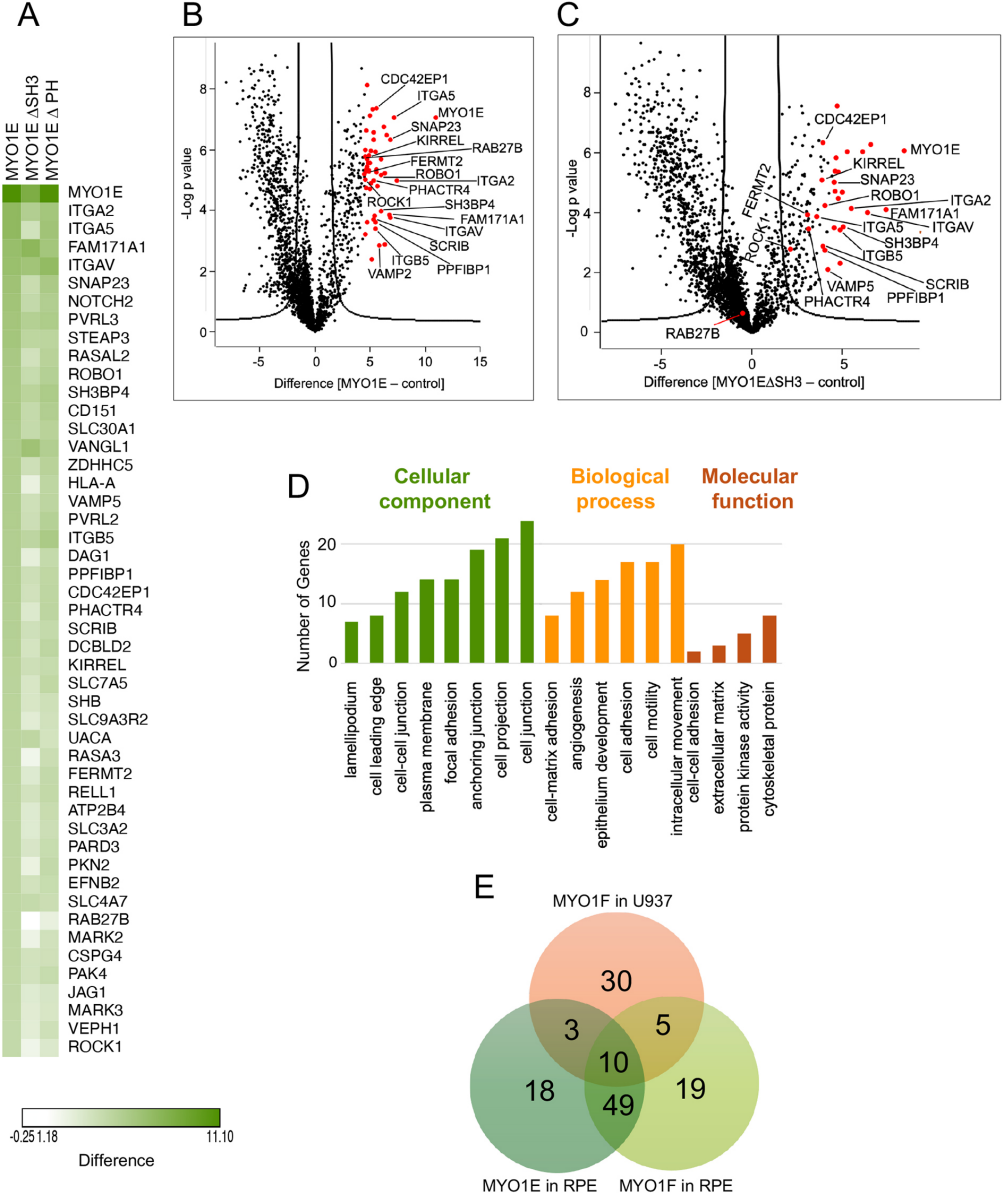
***In situ* proximity labelling reveals new binding partners for MYO1E.** (A) Heat map of abundance of protein interactions with MYO1E tail, MYO1E tail ΔSH3 or MYO1E tail PHmut (ΔPH) determined by BioID from RPE stable cell lines. The heatmap was generated using Morpheus software (https://software.broadinstitute.org/morpheus). Shown are protein interactions with enrichment of 4.5-fold or greater in the BirA*–MYO1E tail experiments over the BirA*-only control. Data are shown from three independent experiments for MYO1E tail, and three independent experiments for each of the two mutants. (B) Volcano plot of LFQ data from BirA*–MYO1E tail over BirA* control from RPE stable cell lines. The negative logarithmic *P*-value was plotted against the *t*-test difference (*n*=3). The hyperbolic cutoff curve delimitates significantly enriched proteins from common hits [FDR of 0.01 and artificial within groups variance of 2]. The complete list is given in Table S2. (C) Volcano plot of LFQ data from BirA*–MYO1E tail ΔSH3 over BirA* control. The negative logarithmic *P*-value was plotted against the *t*-test difference (*n*=3). The hyperbolic cutoff curve delimitates significantly enriched proteins from common hits (FDR of 0.01 and SO of 2). In B and C, significantly enriched and other notable proteins are marked by red dots and labelled by name. (D) GO classification of MYO1E interactome identified by *in situ* proximity labelling. GO cellular component, biological process and molecular function enrichment analyses were performed using ShinyGO software (Ge et al., 2020). A term with *P*<0.05 was considered to be significantly overrepresented. The graph shows the number of proteins in the MYO1E interactome annotated with each significantly overrepresented term. (E) Venn diagram showing the relative numbers of significant interacting proteins detected for MYO1F tail in U937 cells, MYO1F tail in RPE cells and MYO1E tail in RPE cells in three independent BioID experiments. The overlap displays those proteins that were found in multiple constructs. The Venn diagram was made by using the web page http://bioinformatics.psb.ugent.be/webtools/Venn/. The list of proteins represented by the Venn diagram are shown in Table S3.

Proteins with a 4.5-fold or greater enrichment in the MYO1E tail construct over the BirA*-only control were considered significantly enriched. As shown in the heatmap in Fig. 2A, a distinct set of MYO1E-associated proteins was identified, some of which overlapped with the MYO1F interactome. Volcano plot analysis (Fig. 2B) revealed 45 high-confidence MYO1E-interacting proteins, including known actin-binding and membrane-associated proteins such as ITGA5 (Kuninty et al., 2019), FERMT2 (also known as kindlin-2) (Lai-Cheong et al., 2010) and CDC42EP1 (Farrugia and Calvo, 2016). Disruption of the SH3 domain significantly altered the interactome, as illustrated in Fig. 2C and Table S2, indicating that this domain is critical for specific protein interactions. In contrast, mutation of the PH domain had very little effect on the interactome (Fig. 2A; Table S2).

To further explore the potential functional implications of the MYO1E interactome, we performed GO enrichment analysis. Proteins significantly enriched in the MYO1E interactome were associated with actin cytoskeleton organisation, membrane ruffling and cell–substrate junction assembly (Fig. 2D). This supports the notion that MYO1E plays a role in cell adhesion and cytoskeletal remodelling in epithelial cells, consistent with its known role in podosome function (Ouderkirk and Krendel, 2014; Zhang et al., 2019).

Comparison of the interactomes of MYO1E in RPE cells with MYO1F in both U937 and RPE cells revealed overlapping and distinct protein networks (Fig. 2E; Table S3). A total of ten proteins were shared among all three datasets, whereas 30 proteins were unique to MYO1F in U937 cells, 18 were specific to MYO1E in RPE cells and 19 were specific to MYO1F in RPE cells. Interestingly, whereas only three proteins were shared between MYO1F in U937 cells and MYO1E in RPE cells, this number was 49 when the proximity ligation experiments were performed for both myosins in RPE cells, suggesting cell type-specific binding partners for MYO1F. These findings highlight both the conserved and divergent roles of long-tailed class I myosins across different cell types and suggest that MYO1E and MYO1F might perform overlapping yet distinct functions through differential binding partner selection.

### Validation of the CASS group of proteins as a MYO1F-associated adaptor protein complex

**Fig. 3.**
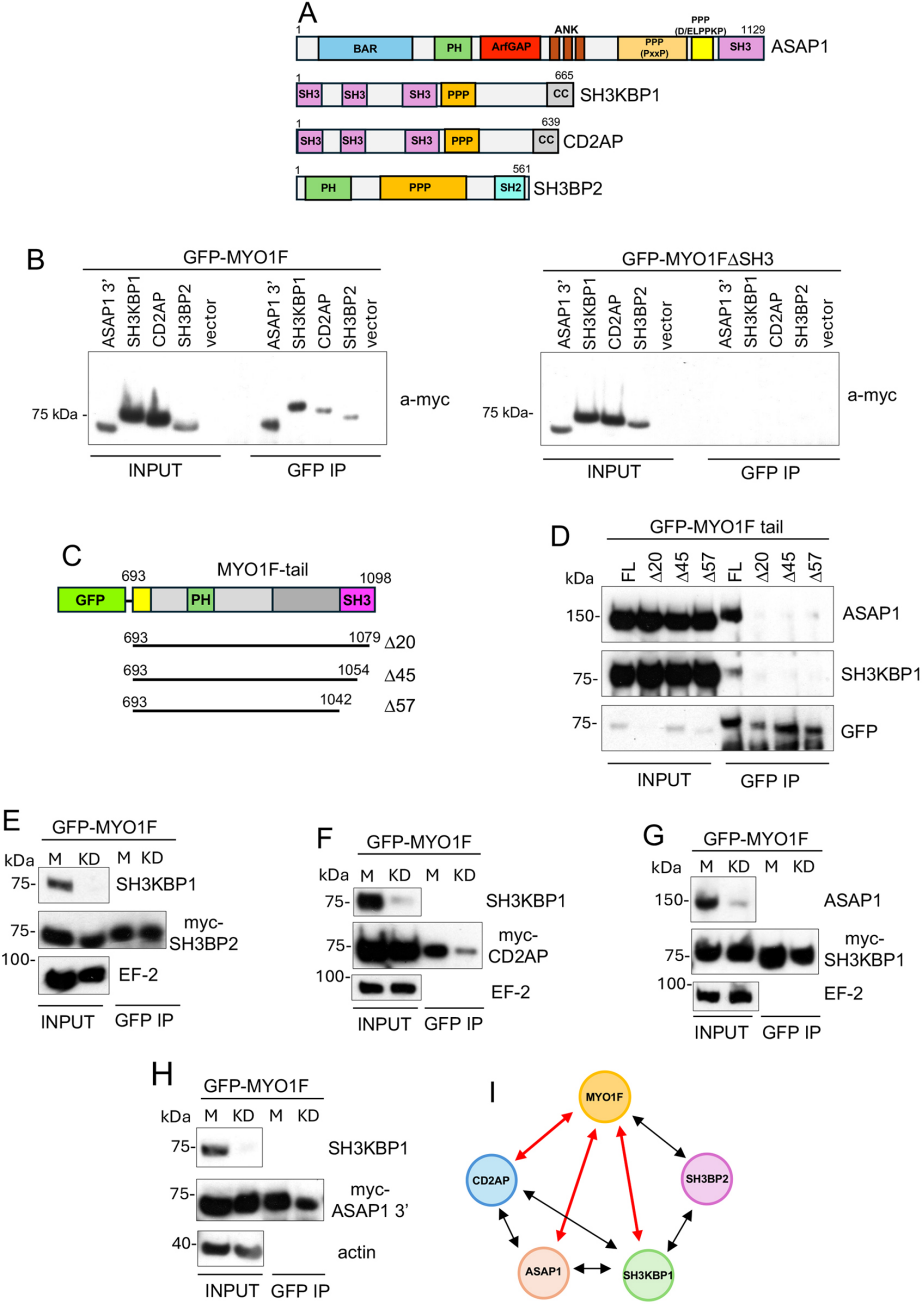
**Verifying the CASS group of proteins as part of the MYO1F interactome by immunoprecipitation.** (A) Schematic cartoon of ASAP1, SH3KBP1, CD2AP and SH3BP2 domain organisation. ANK, ankyrin repeats; BAR, Bin-Amphiphysin-Rvs domain; CC, coiled-coil domain; PPP, proline-rich region; SH2, Src homology 2 domain. (B) Immunoprecipitations (IP) were performed using anti-GFP antibodies from RPE cells stably expressing GFP–MYO1F (left panel) or GFP–MYO1FΔSH3 (right panel) and transiently transfected with Myc–ASAP-3′ (amino acids 589–1129), Myc–SH3KBP1, Myc–CD2AP, Myc–SH3BP2 or empty Myc control vector. Input (4% total) and immunoprecipitates were analysed by western blotting with anti-Myc (a-myc) antibodies. Blots shown are representative of three experiments. (C) Schematic representation of the MYO1F tail domain highlighting the 20, 45 and 57 amino acid deletions at the C terminus of the SH3 domain. (D) Immunoprecipitations were performed from RPE cells transiently expressing either full-length (FL) GFP–MYO1F tail or C-terminal deletions of 20, 45 and 57 amino acids using antibodies to GFP. Immunoprecipitates were analysed by western blotting with antibodies to endogenous ASAP1 or SH3KBP1. Input, 4% total. Blots shown are representative of three experiments. (E) Immunoprecipitations were performed with anti-GFP antibodies from GFP–MYO1F-expressing RPE cells either mock transfected (M) or transfected with siRNA oligonucleotides to SH3KBP1 (KD) before transfection with Myc–SH3BP2. Immunoprecipitates were analysed by western blotting with antibodies to endogenous SH3KBP1 and Myc. EF2 was used as a loading control. (F) GFP–MYO1F was immunoprecipitated from RPE cells either mock transfected (M) or transfected with siRNA oligonucleotides to SH3KBP1 (KD) before transfection with Myc–CD2AP. Immunoprecipitates were analysed by western blotting with antibodies to endogenous SH3KBP1 and Myc. EF2 was used as a loading control. (G) GFP–MYO1F was immunoprecipitated from RPE cells either mock transfected (M) or transfected with siRNA oligonucleotides to ASAP1 (KD) before transfection with Myc–SH3KBP1. Immunoprecipitates were analysed by western blotting with antibodies to endogenous ASAP1 and Myc. EF2 was used as a loading control. (H) GFP–MYO1F was immunoprecipitated from RPE cells either mock transfected (M) or transfected with siRNA oligonucleotides to SH3KBP1 (KD) before transfection with Myc–ASAP1-3′. Immunoprecipitates were analysed by western blotting with antibodies to endogenous SH3KBP1 or Myc. Actin was used as a loading control. In E–H, input lanes show 4% of the total lysate; blots shown are representative of three experiments. (I) Interaction network diagram of the CASS group of proteins. Black arrows highlight known interactions and red arrows show confirmed direct interactions with MYO1F.

To validate MYO1F-binding partners identified by proximity labelling, we performed co-immunoprecipitation (co-IP) assays with GFP-tagged MYO1F and candidate adaptor proteins in RPE cells. Among the most enriched hits in the MYO1F interactome were components of the CASS group of proteins: CD2AP, ASAP1, SH3BP2 and SH3KBP1. These proteins are characterised by multiple SH3 domains, proline-rich regions or PH domains (Fig. 3A), suggesting the potential for multivalent interactions with MYO1F. In GFP pulldown experiments from RPE cells expressing both GFP–MYO1F and Myc-tagged versions of each candidate, we observed co-precipitation of all four proteins with full-length MYO1F (Fig. 3B, left panel; Fig. S4). In contrast, deletion of the SH3 domain (MYO1FΔSH3) abolished binding of CD2AP, SH3KBP1, SH3BP2 as well as ASAP1, indicating that these interactions are dependent on the SH3 domain (Fig. 3B, right panel).

**Fig. 4.**
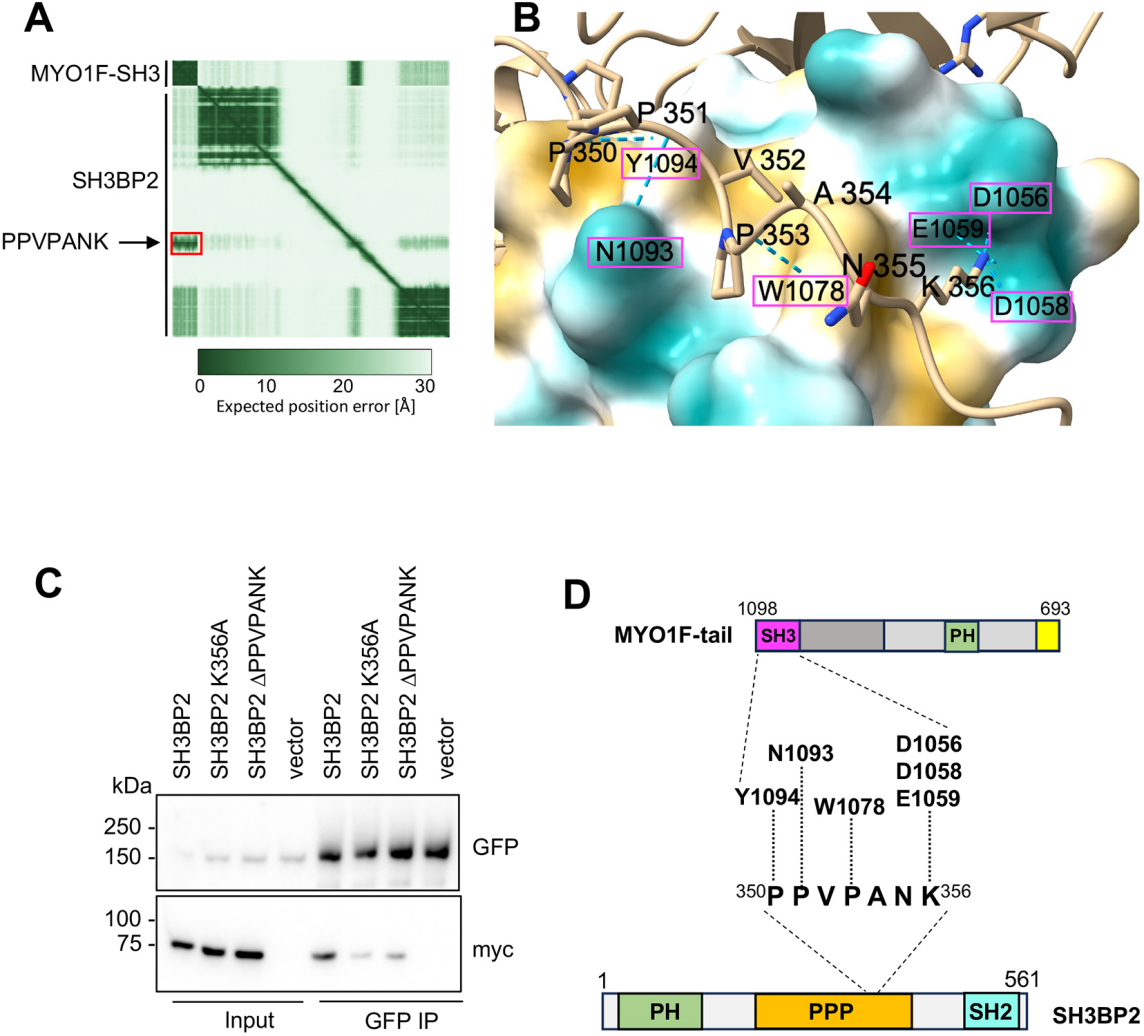
**Characterisation of the molecular interaction between MYO1F SH3-domain and the SH3BP2 proline-rich domain.** (A) Alphafold3-predicted interaction between MYO1F SH3 domain (amino acids 1042–1098) and full-length SH3BP2 (amino acids 1–651). The predicted aligned error (PAE) plot reveals a high-confidence interaction between the proline-rich region of SH3BP2 (PPVPANK, boxed in red) and the MYO1F SH3 domain. The green gradient in the PAE plot indicates confidence levels, with darker regions representing higher prediction confidence. (B) AlphaFold3-predicted structural model showing the interaction interface between the SH3BP2 PPVPANK motif (amino acids 350–356, shown as beige stick structure) and part of the MYO1F SH3 domain (interacting amino acids highlighted by pink boxes). The PPVPANK motif is positioned within a hydrophobic region of the MYO1F SH3 domain (highlighted in yellow). Predicted hydrogen bonding is shown by dashed blue lines. (C) Immunoprecipitation (IP) from RPE cells stably expressing GFP–MYO1F and transiently expressing Myc-tagged SH3BP2 constructs (wild-type, K356A point mutant or a deletion mutant lacking the PPVPANK motif) using GFP-specific nanobodies. Immunoprecipitations were analysed by immunoblotting with antibodies against GFP and Myc. Input, 4%. Blots shown are representative of three experiments. (D) Schematic summary of the predicted molecular interaction between SH3BP2 and the MYO1F SH3 domain, emphasising the role of the PPVPANK motif in binding. PPP, proline-rich region; SH2, Src homology 2 domain.

To analyse the molecular basis of the interaction between MYO1F and the CASS group of proteins, we next generated GFP-tagged MYO1F tail constructs with deletions of 20, 45 and 57 amino acids at the C terminus of the SH3 domain (Fig. 3C). The SH3 domain of MYO1F comprises 58 amino acids and adopts a canonical fold characterised by a compact β-barrel structure formed by five antiparallel β-strands and a short 3₁₀ helix. SH3 domains typically recognise proline-rich motifs with the consensus XPxXP, where X is a hydrophobic residue and x any amino acid. These motifs engage hydrophobic grooves within the SH3 domain via conserved proline residues. Binding specificity is often enhanced by a third, negatively charged pocket that interacts with flanking positively charged residues. Co-IP from transiently transfected RPE cells revealed that deletion of the terminal 20 amino acids markedly reduced MYO1F binding to SH3KBP1 and ASAP1 (Fig. 3D). This region includes N1093 and Y1094, which are predicted by AlphaFold3 (Abramson et al., 2024) to contribute to the interaction with the proline-rich region of SH3BP2 (Fig. 4C,D).

We performed short interfering RNA (siRNA)-mediated knockdowns of SH3KBP1 and ASAP1 before pulling down GFP–MYO1F to further dissect the architecture of the CASS group of proteins. Knockdown of SH3KBP1 had no effect on the binding of SH3BP2 to MYO1F, indicating direct interaction (Fig. 3E). However, knockdown of SH3KBP1 reduced interaction of CD2AP with MYO1F (Fig. 3F), indicating some direct binding of CD2AP to MYO1F and some indirect binding via SH3KBP1. Knockdown of ASAP1 did not reduce SH3KBP1 co-precipitation with MYO1F (Fig. 3G) and vice versa (Fig. 3H), suggesting that both SH3KBP1 and ASAP1 can bind directly to MYO1F.

These results confirm the physical association of MYO1F with a multi-protein complex comprising CD2AP, ASAP1, SH3BP2 and SH3KBP1. The interaction network (Fig. 3I) reflects both previously known interactions (shown by black arrows) and newly established interactions between MYO1F and ASAP1 as well as between MYO1F and SH3KBP1 and CD2AP (highlighted by red arrows). The SH3 domain of MYO1F enables multivalent binding to proline-rich regions found in all members of the CASS group of proteins. Except for SH3BP2, each of these members also contains at least one SH3 domain, facilitating further network formation within the complex.

### *In vivo* experiments confirm the AlphaFold3-predicted binding interface between the proline-rich domain of SH3BP2 and the MYO1F-SH3 domain

To further explore the SH3-domain–ligand interface, we used AlphaFold3 to model the interaction between the MYO1F SH3 domain (amino acids 1042–1098) and full-length SH3BP2 (amino acids 1–651). While MYO1F has been previously identified as a binding partner for SH3BP2 (Navines-Ferrer et al., 2019), no molecular detail on the binding interface has been established so far. To determine the exact interaction sites between SH3BP2 and MYO1F, we used Alphafold3 modelling, which predicts a high-confidence interaction (ipTM=0.87), shown as a red boxed area on the predicted aligned error (PAE) plot, between the MYO1F SH3 domain and a short proline-rich motif in SH3BP2 (PPVPANK; amino acids 350–356) (Fig. 4A). The ipTM score provides an estimate of the predicted accuracy of the relative positioning between interacting protein domains, with values above 0.8 indicating high-confidence, high-accuracy predictions, whereas scores between 0.6 and 0.8 represent lower-confidence models that may be either correct or incorrect. The SH3BP2 proline-rich motif fits into a hydrophobic groove on the MYO1F SH3 domain, with multiple hydrogen bonds predicted to stabilise the interaction (Fig. 4B,D). Structural modelling revealed that prolines P350 and P351 in SH3BP2 bind to residues Y1094 and N1093 in the MYO1F SH3 domain, whereas P353 interacts with W1078. In addition, the terminal lysine (K356) of the motif docks into a negatively charged specificity pocket formed by D1056, D1058 and E1059 of MYO1F (Fig. 4B,D).

To validate this predicted binding interface in cells, we generated Myc-tagged SH3BP2 constructs with either a point mutation of the predicted core lysine (K356A) or a deletion of the entire PPVPANK motif (ΔPPVPANK). These constructs were co-expressed with GFP–MYO1F in RPE cells, and GFP-based pulldowns performed. Western blotting revealed that both the K356A substitution and the PPVPANK deletion significantly reduced the interaction with MYO1F, confirming the functional importance of this motif (Fig. 4C). Interestingly, deleting the PPVPANK motif did not fully abolish the interaction with MYO1F, suggesting that the additional proline-rich sequences present within SH3BP2 might allow interaction with the SH3 domain.

Taken together, these findings provide structural and biochemical evidence that the MYO1F SH3 domain recognises the PPVPANK motif in SH3BP2 through canonical SH3 domain interactions, involving two hydrophobic grooves and a specificity pocket. The PPVPANK motif is thus the principal determinant for high-affinity SH3-domain-mediated binding (Fig. 4D).

### Identification of multiple proline-rich motifs in ASAP1 that mediate binding to the MYO1F SH3 domain

**Fig. 5.**
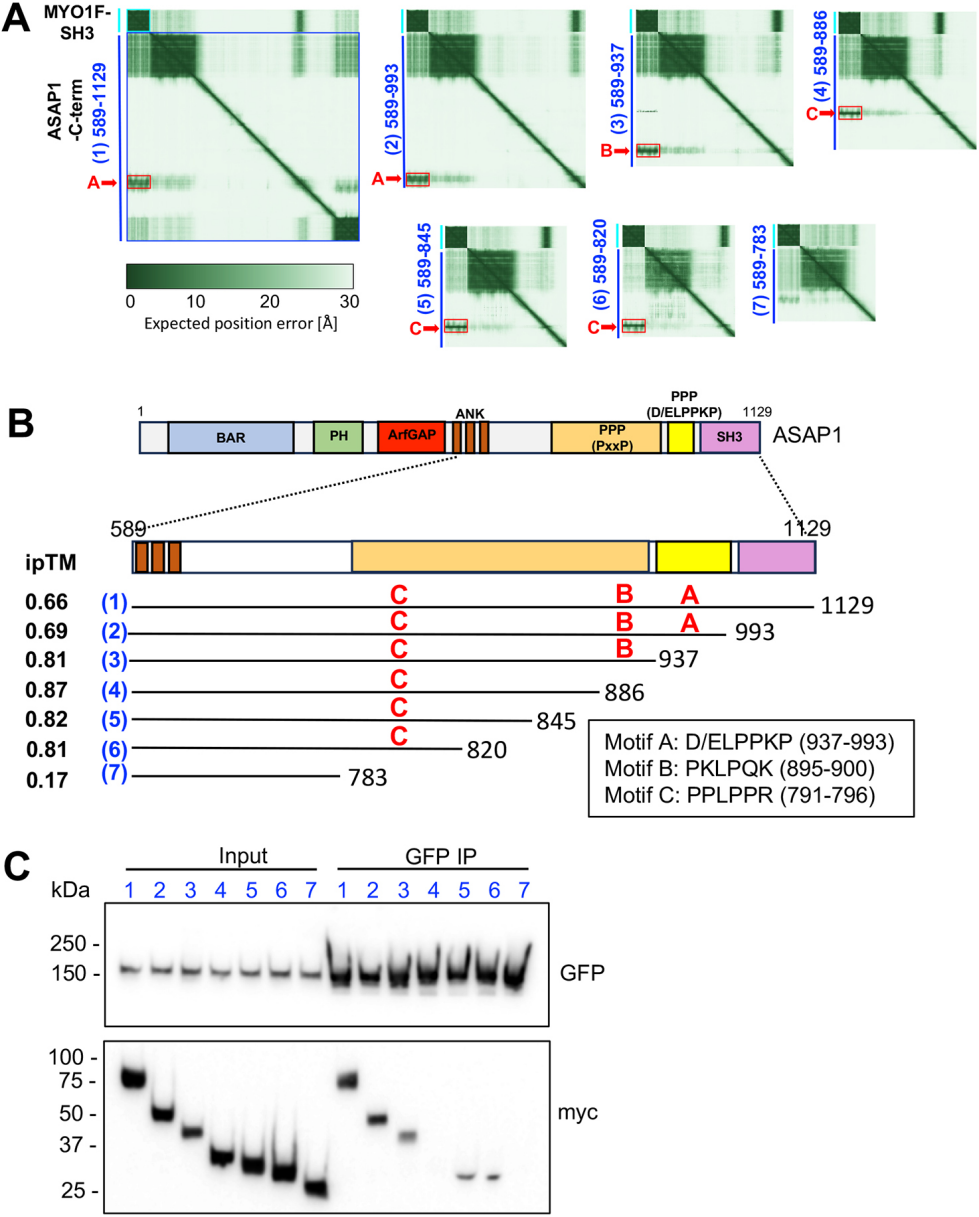
**Experimental verification of the predicted MYO1F–ASAP1 interaction.** (A) AlphaFold3-predicted interaction of MYO1F with the C terminus of ASAP1 (amino acids 589–1129) and six truncation constructs spanning the indicated amino acid residues. Each PAE plot highlights intramolecular confidence of interaction, where darker green represents higher confidence. Red boxes denote predicted binding regions aligning with conserved proline-rich motifs (designated A, B and C). Constructs are numbered 1–7 with reducing lengths. (B) Schematic illustration of full-length ASAP1, illustrating key domains: BAR (Bin-Amphiphysin-Rvs), PH, ANK (ankyrin repeats), PPP (proline-rich region; PxxP or D/ELPPKP repeats) and SH3. Enlarged C terminus of ASAP1 (amino acids 589–1129) with overview of ASAP1 truncation constructs (1–7 in blue) tested for MYO1F binding underneath. The proline-rich domain of ASAP1 contains several proline-rich motifs (designated A, B and C) that are predicted by AlphaFold3 to bind the MYO1F SH3 domain. Proline-rich motifs are labelled A, B or C in red, with their predicted ipTM score on the left-hand side. (C) Immunoprecipitation (IP) from RPE cells stably expressing GFP–MYO1F and transiently expressing Myc-tagged ASAP1 truncation constructs (1–7, as in A,B). GFP pulldowns were performed using GFP-specific nanobodies. Input (4% total) and immunoprecipitated fractions were analysed by western blotting with anti-Myc and anti-GFP antibodies. Blots shown are representative of three experiments.

To further dissect the molecular interface between MYO1F and ASAP1, we also used AlphaFold3 to model the interaction between the MYO1F SH3 domain and the C-terminal half of ASAP1 (amino acids 589–1129). Structural predictions revealed several proline-rich motifs in ASAP1 that could potentially bind the MYO1F SH3 domain, with distinct confidence scores indicated by ipTM values (Fig. 5A,B). In the D/ELPPKP domain, several PPKP motifs between residues 937 and 993 (Fig. 5B, region A in constructs 1 and 2) with lower-confidence ipTM scores between 0.66 and 0.69 were identified. Consistent with the lower ipTM scores, co-IP assays using GFP–MYO1F and Myc-tagged ASAP1 truncation constructs without these motifs showed only slight reduction in MYO1F binding (Fig. 5C, see construct 3), suggesting that these predicted motifs are not essential for the observed interaction.

To map the minimal region of ASAP1 required for MYO1F interaction, further C-terminal truncations, in combination with AlphaFold3 predictions, were generated between residues 589 and 937 and then assessed for MYO1F binding (Fig. 5C). Whereas deletion of the class II PKLPQK motif (amino acids 895–900, motif B), does not completely abolish ASAP1–MYO1F interaction (Fig. 5C, see constructs 5 and 6), deletion of residues 784–820 abrogated MYO1F binding (Fig. 5C, see construct 7), suggesting that a proline-rich motif within this region mediates binding. AlphaFold3 predicted a class II PPLPPR motif (amino acids 791–796, motif C) in this segment with an ipTM of 0.87. Supporting this prediction, deletion of this region abolished binding (Fig. 5C, see construct 7), whereas its inclusion in constructs 5 and 6 resulted in retention of the MYO1F interaction. Constructs 4 appears to be an anomaly, as binding to MYO1F was abolished (Fig. 5C) despite this construct retaining the putative binding site in motif C. This observation might suggest that intramolecular folding or structural context also influence motif accessibility or binding affinity.

In summary, AlphaFold3 predictions together with experimental truncation mapping highlight that the MYO1F SH3 domain has the potential to interact with multiple proline-rich motifs in the C terminal region of ASAP1. These results suggest that MYO1F exhibits binding plasticity across several partially redundant proline-rich motifs, potentially allowing motor multimerisation.

### Subcellular localisation of MYO1F and its adaptor proteins in THP-1 macrophages

**Fig. 6.**
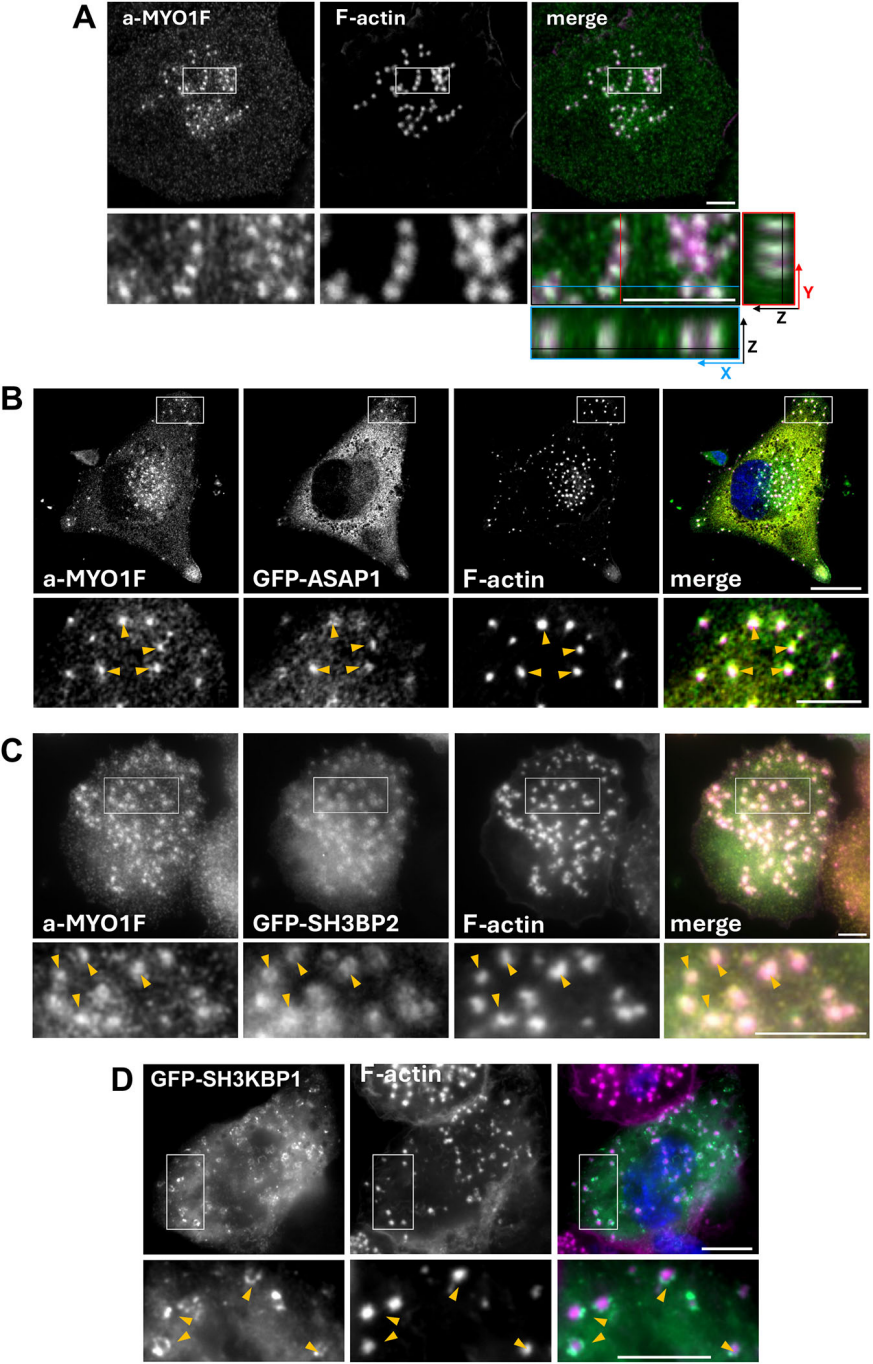
**MYO1F and ASAP1 colocalise in ventral podosomes in macrophages.** (A) THP-1 cells treated with PMA to induce differentiation into adherent macrophage-like cells were stained by immunofluorescence with antibodies to MYO1F (a-MYO1F) and double labelled with fluorescently conjugated phalloidin to visualise F-actin. In the merge image, actin is shown in magenta and MYO1F in green. Enlarged *XY* views are shown below the main images. Images below and to the right of the enlarged merge *XY* view show the *XZ* view along the blue line and *ZY* view along the red line, respectively. (B,C) THP-1 cells were transiently transfected with GFP–ASAP1 (B) or GFP–SH3BP2 (C), labelled with antibodies to GFP and MYO1F (a-MYO1F), and stained with fluorescently conjugated phalloidin to visualise F-actin. The merge images show actin in magenta, GFP–ASAP1 (B) or GFP–SH3BP2 (C) in green, a-MYO1F in yellow and the nucleus in blue. Confocal images were taken at the dorsal layer of the cell close to the coverslip in A, B and C. (D) THP-1 cells transiently expressing GFP–SH3KBP1 were labelled with antibodies to GFP and fluorescently conjugated phalloidin to visualise F-actin. The merge image on the right shows actin in magenta, GFP–SH3KBP1 in green and the nucleus in blue. Widefield images focused on the dorsal part of the cell are shown. White boxes in A–D indicate areas enlarged in the pictures below. Orange arrowheads indicate colocalisation. Scale bars: 5 µm. Images shown are representative of three experiments.

We next examined the subcellular localisation of endogenous MYO1F and its validated direct binding partners, ASAP1, SH3KBP1 and SH3BP2, in myeloid cells using high-resolution immunofluorescence microscopy in phorbol 12-myristate 13-acetate (PMA)-differentiated THP-1 macrophages. Confocal microscopy of cells stained with anti-MYO1F antibodies and phalloidin revealed that MYO1F localises to discrete actin-rich puncta at the ventral cell surface that are surrounded by a ring of plaque proteins such as vinculin, consistent with the organisation of podosomes (Fig. 6A; Fig. S5) (Linder et al., 2023). These MYO1F-positive puncta were spatially restricted to the basal cortex, as shown in the *z*-plane proximal to the coverslip (Fig. 6A). Although we did observe some variability in the precise distribution of endogenous MYO1F between different cell types and occasionally between individual cells within the same experiment, the predominant pattern we observed was localisation with F-actin in the podosome core (see *xz* view in Fig. 6A). Our observations confirm the previously reported identification of MYO1F as a podosome component by detailed microscopy and mass spectroscopy (Cervero et al., 2025, 2012). Furthermore, both ASAP1 and MYO1E, the closest mammalian homologue of MYO1F, have been localised at podosomes (Bharti et al., 2007; Ouderkirk and Krendel, 2014). Whereas ASAP1 has been implicated in podosome formation and turnover via interactions with proteins such as GEFH1 (also known as ARHGEF2; Shiba and Randazzo, 2011) and FAK (also known as PTK2; Bharti et al., 2007), MYO1E has been suggested to regulate actin polymerisation and membrane–cytoskeleton coupling in a phosphatidylinositol (3,4,5)-trisphosphate-dependent manner (Zhang et al., 2019).

To further compare the spatial distribution of MYO1F and its binding partners ASAP1 and SH3BP2, we co-stained THP-1 macrophages transiently expressing GFP–ASAP1 or GFP–SH3BP2 with anti-MYO1F antibodies and phalloidin. Confocal imaging revealed colocalisation of GFP–ASAP1 and endogenous MYO1F, and of GFP–SH3BP2 and MYO1F, at ventral actin puncta (Fig. 6B,C), indicating that MYO1F and its binding partners ASAP1 and SH3BP2 are recruited to podosomes. Finally, GFP–SH3KBP1 exhibited punctate localisation patterns, similar to MYO1F, that overlapped with phalloidin-labelled F-actin (Fig. 6D), indicating recruitment to podosomes. Enlarged views highlighted that SH3KBP1 and SH3BP2 appear not to completely overlap with the actin core of podosomes but rather form ring-like structures, which might represent their association with distinct sub-domains of the podosome, such as the surrounding adhesion plaque or regulatory complexes at the periphery.

These observations confirm that MYO1F and members of the CASS group of proteins (ASAP1, SH3KBP1 and SH3BP2) are recruited to actin-rich podosomes in macrophages, supporting a functional role for this protein complex in cytoskeletal organisation and membrane–cytoskeleton coupling.

### MYO1F and ASAP1 colocalise at podosomes in primary mouse microglia and iPSC-derived human microglia

Given the upregulation of MYO1F expression in microglia within both murine models of neurodegeneration and tissue from individuals with AD (Gao et al., 2023; Matarin et al., 2015; Mukherjee et al., 2019; Zhang et al., 2013), we examined the subcellular localisation of endogenous MYO1F and its adaptor protein ASAP1 in both primary mouse microglia and human iPSC-derived microglia to assess whether recruitment to podosomes is conserved across different myeloid cell types.

**Fig. 7.**
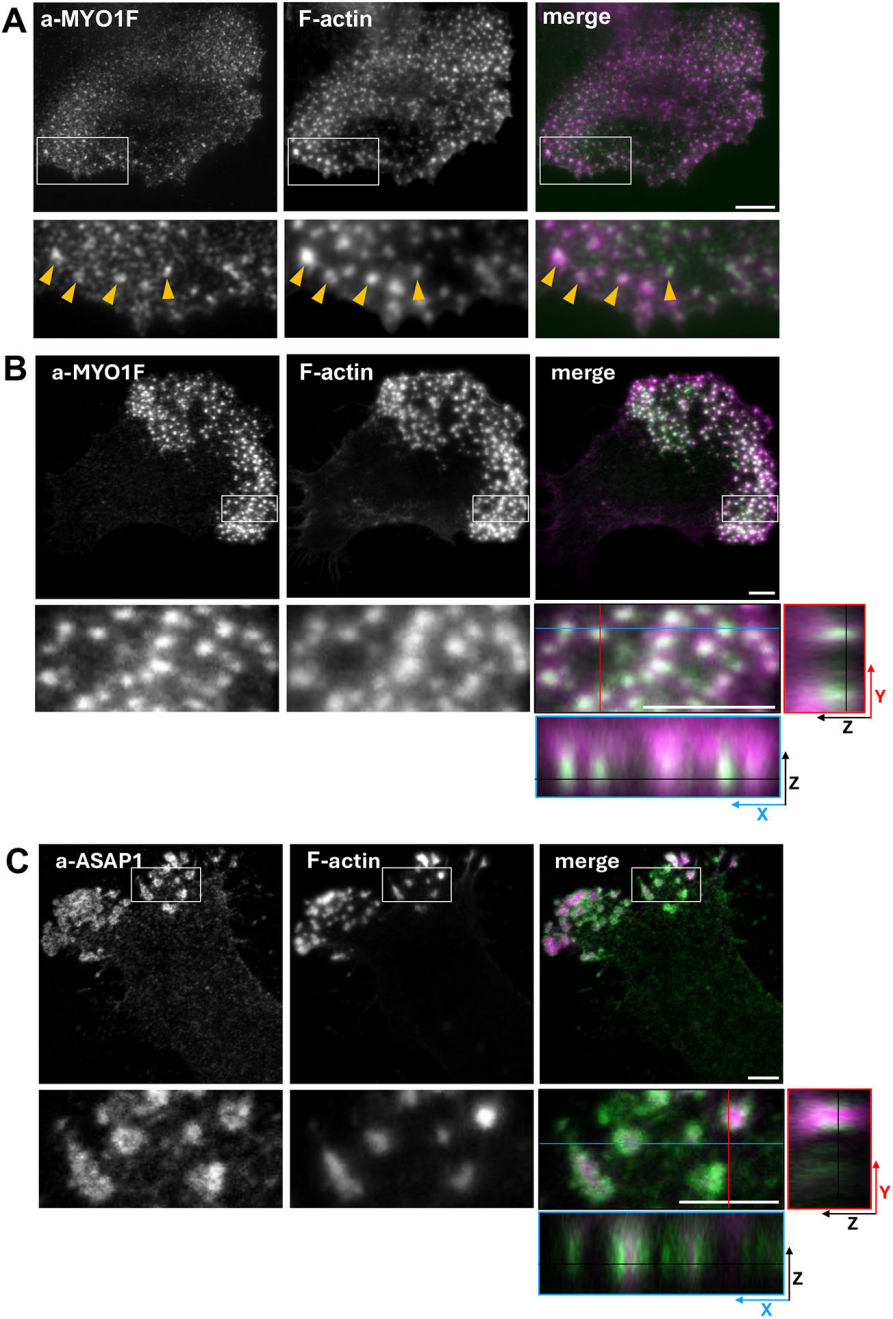
**MYO1F and ASAP1 colocalise in podosomes in primary mouse microglia and iPSC-derived human microglia.** (A) Primary mouse microglia on coverslips were stained with antibodies to MYO1F (a-MYO1F) and labelled with fluorescently conjugated phalloidin to visualise F-actin. The merge image shows actin in magenta and MYO1F in green. White boxes indicate areas enlarged in the pictures below. Orange arrowheads indicate colocalisation. Widefield images focused on the dorsal part of the cell are shown. Scale bar: 5 µm. (B,C) Human iPSC-derived microglia were stained with antibodies to MYO1F (B, a-MYOF1) or ASAP1 (C, a-ASAP1) and labelled with fluorescently conjugated phalloidin to visualise F-actin. The merge images on the right show actin in magenta, MYO1F (B) or ASAP1 (C) in green. White boxes indicate areas enlarged in the pictures below. Images below and to the right of the enlarged merged *XY* views show the *XZ* view along the blue line and *ZY* view along the red line, respectively. Confocal images were taken at the dorsal layer of the cell close to the coverslip. Scale bars: 5 µm. Images shown are representative of three experiments.

Immunofluorescence microscopy of primary mouse microglia revealed that MYO1F localises to discrete phalloidin-labelled F-actin structures at the ventral surface of the cell, suggesting the recruitment of MYO1F to podosomes in brain-derived microglia (Fig. 7A).

To determine whether these observations extend to human microglia, we assessed the localisation of MYO1F and ASAP1 in human iPSC-derived microglia, which express microglia marker proteins such as the ionized calcium-binding adapter molecule 1 (IBA-1, also known as AIF1), the purinergic receptor (P2Y12, also known as P2RY12) and the transmembrane protein 119 (TMEM119) (Devlin et al., 2025; Stoessel et al., 2025; Uff et al., 2022) (Fig. S6). Confocal imaging revealed that both MYO1F and ASAP localise to punctate structures at the basal cortex, colocalising with F-actin-rich domains. In addition, ASAP1 formed ring-like structures that surrounded the F-actin puncta (Fig. 7B,C). These structures stained positive for the podosomal markers vinculin, cortactin and talin (Fig. S7), and were morphologically consistent with podosomes observed in peripheral myeloid cells and primary mouse microglia (Figs 6 and 7A).

These findings confirm the localisation of MYO1F and ASAP1 at the core and periphery of podosomes, respectively, not only in macrophages but also in mouse and human microglia. Given the known involvement of podosomes in adhesion and matrix remodelling (Alonso et al., 2019), and the reported transcriptional upregulation of MYO1F in neuroinflammatory states associated with neurodegenerative disease (Matarin et al., 2015; Zhang et al., 2013), the conserved podosomal recruitment of MYO1F and its adaptor protein ASAP1 supports a role for this complex in cytoskeletal remodelling and immune function within the central nervous system.

### MYO1F recruitment to the phagocytic cup requires motor activity and adaptor proteins

To investigate the mechanisms underlying MYO1F localisation during phagocytosis, we examined the recruitment of MYO1F and its known adaptor proteins to phagocytic cups. Both MYO1F and its paralogue MYO1E are enriched at the phagocytic cup, where they regulate cortical tension and promote phagosome closure (Barger et al., 2019, 2022; Maxeiner et al., 2015; Vorselen et al., 2021). Interestingly, podosome-like structures, called phagocytic podosomes, can also be observed at the ventral edge of the phagocytic cup (Herron et al., 2022). These structures might function as adhesion sites that generate traction for particle internalisation.

**Fig. 8.**
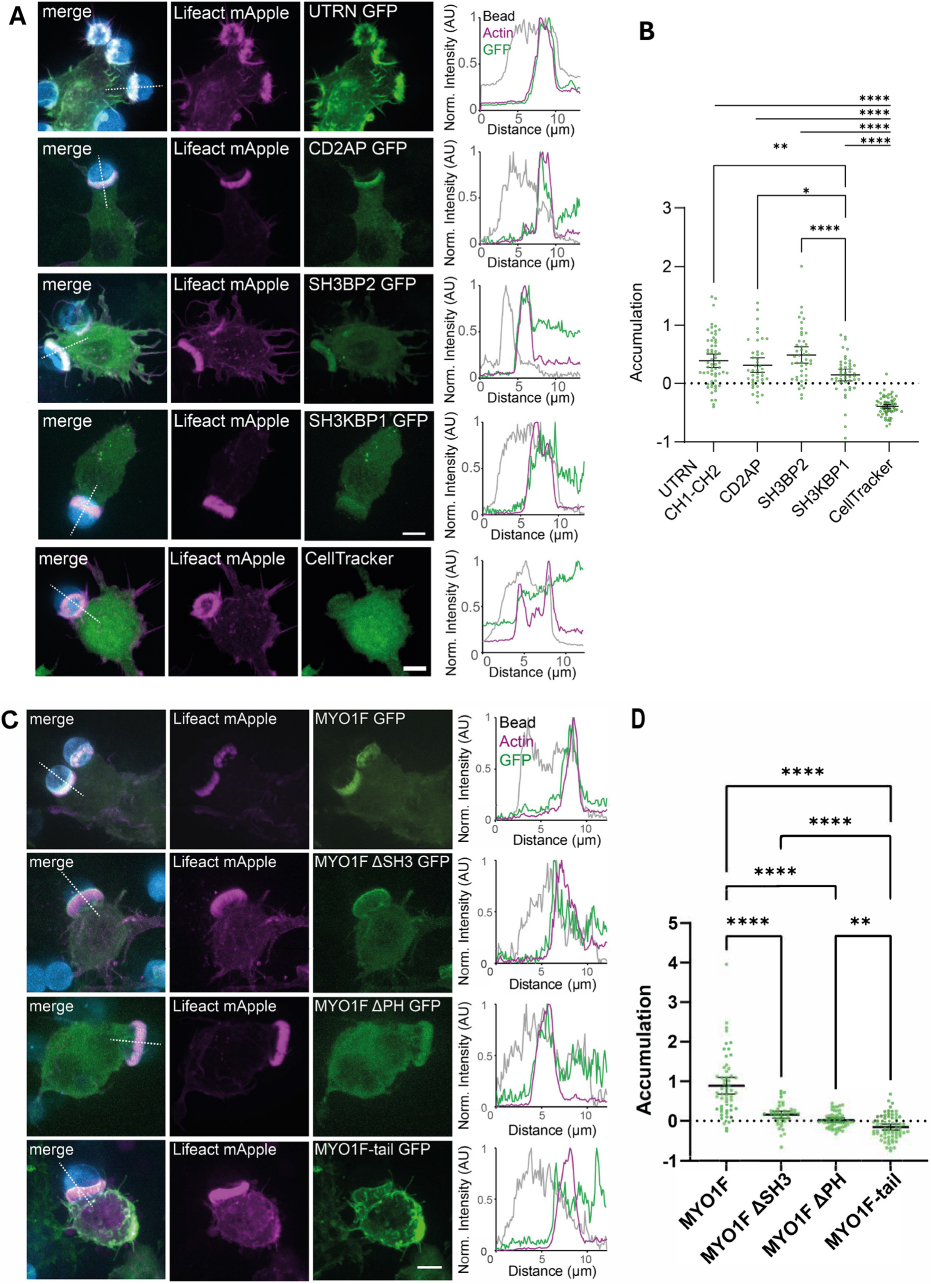
**Recruitment of MYO1F and adaptor proteins to the phagocytic cup.** (A) Fluorescence images of RAW246.7 cells expressing Lifeact–mApple (magenta) and the GFP-tagged binding partner of interest (green), engulfing AlexaFluor647-labelled beads (blue). CellTracker is shown as a negative control. Scale bars: 5 µm. Line profiles along the dashed line (white, merge) show the normalised fluorescence intensity of the bead, actin and the protein of interest (right). (B) Dot plot of accumulation values comparing the proteins UTRN CH1-CH2 (*n*=60), CD2AP (*n*=42), SH3BP2 (*n*=44) and SH3KBP1 (*n*=46). Data shown were collected from three independent experiments. Bars represent the mean±95% c.i. ****P<0.0001; **P<0.01; *P<0.05 (two-sided Student's *t*-test). (C) Fluorescence images of RAW246.7 cells expressing Lifeact–mApple (magenta) and GFP-tagged MYO1F, MYO1F ΔSH3, MYO1F PHmut (ΔPH) or MYO1F tail (green). The cells are shown engulfing AlexaFluor647-labelled beads (blue). Scale bar: 5 µm. Line profiles along the dashed line (white, merge) show the normalised fluorescence intensity of the bead, actin and the protein of interest (right). (D) Dot plot of accumulation values comparing cells expressing MYO1F (*n*=55) with MYO1F ΔSH3 (*n*=42), MYO1F PHmut (*n*=56) and the tail of MYO1F (*n*=65). Data shown were collected from three independent experiments. Bars represent the mean±95% c.i. ****P<0.0001; **P<0.01 (unpaired two-sided Student's *t*-test). AU, arbitrary units.

To determine which MYO1F-associated adaptor proteins are recruited to the phagocytic cup, we performed three-colour confocal imaging of RAW264.7 macrophages transfected with Lifeact–mApple and GFP-tagged constructs of MYO1F binding partners (Fig. 8A). Following the addition of AlexaFluor647-labelled bovine serum albumin (BSA)-coated beads to stimulate phagocytosis, we assessed the accumulation of each protein at the actin-rich phagocytic cup (Fig. 8B). In this assay we also included the analysis of the utrophin actin-binding calponin homology domain (UTRN CH1-CH2) as a positive control alongside Lifeact. As UTRN is a very large protein, we used a shorter form containing only the actin-binding calponin homology domains (CH1-CH2). Quantification of the recruitment of the putative binding partners revealed that the UTRN CH1-CH2 domain (0.4±0.44, mean±95% c.i.), CD2AP (0.32±0.41) and SH3BP2 (0.49±0.45) accumulated to a similar extent at the phagocytic cup, whereas SH3KBP1 showed lower enrichment (0.14±0.34) (Fig. 8B). ASAP1 is not expressed in RAW264.7 macrophages and was therefore not included in this assay.

To define the structural features required for MYO1F localisation during phagocytosis, we next compared the recruitment of full-length MYO1F with mutant constructs lacking specific domains using the same assay (Fig. 8C). Full-length MYO1F–GFP displayed robust enrichment at the phagocytic cup (0.89±0.79, mean±95% c.i.; Fig. 8C,D) consistent with a role in linking membrane dynamics to cortical actin during particle engulfment (Barger et al., 2022; Vorselen et al., 2021). In contrast, mutation of the pleckstrin homology domain (ΔPH) or deletion of the SH3 domain (ΔSH3) led to significantly reduced accumulation (0.02±0.20 and 0.16±0.29, respectively). A tail-only construct, MYO1F-tail, which lacks the motor domain, showed no enrichment at the phagocytic cup (−0.16±0.31; Fig. 8C,D).

In summary, our results demonstrate that MYO1F recruitment to the phagocytic cup requires intact motor activity, membrane association via the PH domain and adaptor protein binding via the SH3 domain. Together, these findings provide mechanistic support for the role of MYO1F in bridging membrane–cytoskeleton interactions during phagocytosis and underscore a potential mechanistic convergence between podosome and phagosome biology, as MYO1F functions in both processes where actin-rich, podosome-like structures contribute to membrane remodelling and substrate engagement

## DISCUSSION

In this study we have used *in situ* proximity labelling-based proteomics to identify the interaction network of the two human long-tailed myosins of class I, MYO1E and MYO1F. We determined the interactome for both myosins using cell types in which they are endogenously expressed at high levels: RPE cells for MYO1E and U937 cells for MYO1F. Using a combination of co-IP, mutagenesis, structural modelling and cell biological assays we characterised the functional CASS group of proteins, comprising CD2AP, ASAP1, SH3KBP1 and SH3BP2, which interacts with the C-terminal SH3 domain of MYO1F. The CASS group of proteins is characterised by multiple SH3 and proline-rich motifs in addition to previously established mutual interactions, indicating a pre-assembled adaptor network (Dikic, 2002; Kirsch et al., 1999; Take et al., 2000). This distinguishes the CASS group from other MYO1F SH3-domain-binding proteins, such as WAS, which contains proline-rich motifs but lacks SH3 domains and is not known to interact with any of the CASS components. In addition, we identified a number of membrane-localised proteins that depend on the intact MYO1F PH domain for binding, including NUMB, ADD3, PECAM1, GOLGA8R and GPR124 (also known as ADGRA2).

Together, our proximity labelling and domain deletion strategies reveal two distinct functional modules within the MYO1F interactome: (1) an SH3-domain-dependent group of proteins (CASS), involved in cytoskeletal organisation and adaptor scaffolding, and (2) a PH-domain-associated set of membrane-associated proteins. These modules likely enable MYO1F to form a dynamic link between the plasma membrane and the actin cytoskeleton during processes such as podosome formation and phagocytosis.

Comparison of the interactomes revealed limited overlap between MYO1F in U937 and MYO1E in RPE cells, reflecting their divergent tissue distributions and distinct functions. MYO1F expression is largely restricted to immune cells (Kim et al., 2006; Salvermoser et al., 2018), as confirmed by immunoblotting (Fig. 1A), whereas MYO1E is broadly expressed across a variety of tissues including kidney, lung, intestine and immune cells (Liu et al., 2024; Stöffler et al., 1995). In neutrophils, where both proteins are present at differing levels, MYO1E and MYO1F exhibit non-redundant roles. MYO1E deficiency causes increased rolling velocity, decreased adhesion, aberrant crawling and strongly reduced transmigration through the capillary wall (Vadillo et al., 2019). The highly expressed MYO1F is important for neutrophil migration, as cells from the MYO1F knockout mouse show stronger adhesion to integrin ligands, consequently reducing cell motility under static conditions *in vitro* (Kim et al., 2006). Finally, in mast cells, MYO1F appears to regulate cortical actin filament dynamics affecting cell adhesion, migration and degranulation, a process that involves the adaptor protein SH3BP2, which we identified via *in situ* proximity labelling and verified using mutagenesis guided by AlphaFold-based structural predictions (Navines-Ferrer et al., 2019). The display of these functional differences between MYO1E and MYO1F, when they are expressed in the same cell type, is supported by the only partially overlapping interactome. The limited sequence conservation in the tail region of only 55% identity does allow for distinct adaptor protein binding.

It has previously been demonstrated that the TH2 domain of MYO1E is essential for its podosomal localisation, that the TH1 domain promotes its membrane enrichment and ventral localisation, and that deletion of the SH3 domain does not impair its recruitment to podosomes (Ouderkirk and Krendel, 2014; Zhang et al., 2019). While it has been shown that the SH3 domain is dispensable for MYO1E, corresponding data for MYO1F have not been presented. In contrast, our study reveals that the SH3 domain of MYO1F is essential for binding multiple podosome-resident adaptor proteins, including ASAP1, SH3KBP1 and SH3BP2. These interactions suggest that the SH3 domain of MYO1F is not crucial for recruitment but is important for functional engagement at podosomes and phagocytic structures. Notably, these binding partners are not shared with MYO1E, highlighting a mechanistic divergence from MYO1E and distinct recruitment strategies among long-tailed myosins of class I.

Previous studies have reported localisation of MYO1E and its SH3 domain mutants to phagocytic actin waves during frustrated phagocytosis (Barger et al., 2019). In our bead-based assay, however, we revealed that the MYO1F tail and ΔSH3 mutant do not accumulate at the three-dimensional phagocytic cup. These differences likely arise from the distinct geometry and dynamics of the two assays, with planar actin waves forming on IgG-coated surfaces versus confined actin cups during bead engulfment. Furthermore, MYO1F and MYO1E display only partial sequence conservation in their tail domains and engage distinct adaptor networks, with MYO1F uniquely binding the CASS group of proteins via its SH3 domain. These differences suggest that long-tailed class I myosins, while functionally related, employ divergent adaptor-based mechanisms for recruitment and regulation during phagocytosis.

To date, most functional studies of MYO1F have focused on peripheral immune cells; however, emerging evidence indicates that MYO1F is also expressed in brain-resident microglia, the principal innate immune cells of the central nervous system. Although the precise molecular functions of MYO1F in microglia remain to be elucidated, our immunofluorescence studies in both primary mouse microglia and human iPSC-derived microglia reveal that MYO1F localises to ventral actin-rich structures positive for podosomal marker proteins. These specialised adhesion sites are known to regulate cell–substrate interactions, matrix degradation and mechanotransduction. The recruitment of MYO1F to podosomes suggests that it might contribute to microglial adhesion, migration and possibly cytokine secretion during neuroimmune responses.

There is gathering evidence for the dysregulation of peripheral and innate immune systems in neurodegenerative pathologies, including a skewed susceptibility of myeloid and microglial cells to inflammatory activation (McCauley et al., 2020; Vahsen et al., 2023). Given the selective upregulation of MYO1F in activated microglia in the context of neurodegenerative disorders, MYO1F warrants further investigation as a pharmacological target for regulating microglial activation and attenuating neuroinflammation in conditions such as AD or FTD/ALS.

Importantly, myosin motors are considered druggable proteins, exemplified by the development of small-molecule modulators for several myosin classes (Bond et al., 2013; Manstein and Preller, 2020). Finally, the SH3KBP1–CD2AP complex binding to MYO1F has also been linked to neurodegeneration, particularly AD. CD2AP is an adaptor protein involved in actin and membrane trafficking, and genetic variants of CD2AP have been associated with increased risk for late-onset AD (Hollingworth et al., 2011; Naj et al., 2011). SH3KBP1 is a scaffold protein also involved in endocytosis and receptor tyrosine kinase trafficking (Havrylov et al., 2010). While less studied than CD2AP in neurodegeneration, emerging data implicate SH3KBP1 in neuroinflammatory pathways. Recent work has further demonstrated that CD2AP, similar to MYO1F, is upregulated in microglia during AD, highlighting the possibility that the MYO1F–CD2AP interaction underlies cytoskeletal remodelling and cargo trafficking in reactive microglia, contributing to the cellular reprogramming observed in neurodegenerative diseases.

## MATERIALS AND METHODS

### Antibodies

The following antibodies were used: MYO1F [Santa Cruz Biotechnology Inc. Dallas, TX, USA; sc-376534; 1:500 western blotting (WB), 1:100 immunofluorescence (IF)], MYO1E (Atlas Antibodies, Stockholm, Sweden; HPA023886; 1:500 WB), actin (Sigma-Aldrich, Gillingham, Dorset, UK; A2066; 1:2000 WB), IBA1 [Fujifilm Wako Chemicals Europe, Neuss, Germany; 019-19741; 1:2000 (WB), 1:1000 (IF)], P2Y12 (Biolegend UK Ltd., London, UK; 848004; 1:500), TMEM119 (Novus Biologicals, CO, USA; NBP2-30551; 1:500), GFP (Thermo Fisher Scientific, Waltham, MA, USA; A11122; 1:2000 WB, 1:1000 IF), Myc (Merck Life Sciences UK Ltd.; 05-724; 1:1000 WB, 1:500 IF), SH3KBP1 (Santa Cruz Biotechnology Inc.; sc-166862; 1:500 WB), ASAP1 (Santa Cruz Biotechnology Inc.; sc-374410; 1:500 WB), EF2 (Santa Cruz Biotechnology Inc.; sc-166415; 1:1000), vinculin (Sigma-Aldrich; V4505; 1:100), talin (Abcam, Cambridge, UK; ab11188; 1:100), cortactin [Santa Cruz Biotechnology Inc.; sc55579; 1:50 (IF)]. All secondary antibodies for immunofluorescence were purchased from Thermo Fisher Scientific.

### Plasmids

The MYO1F and CD2AP cDNAs were amplified by PCR from THP-1-derived cDNA, whereas the MYO1E tail domain and ASAP1 were amplified from RPE cDNA. SH3BP2 was amplified from HeLa cDNA, and SH3KBP1 (isoform 1) from U937 cDNA. Full-length or tail constructs were generated by PCR and subcloned into pEGFP-C or pCMV-myc (both our laboratory stocks; available upon request), The pcDNA3.1 myc-BioID vector was obtained from Addgene (Addgene #35700) and subcloned into the pLXIN2 retroviral vector (Addgene #99203). GFP-pLXIN2 was generated by replacing myc-BirA from myc-BirA pLXIN2 with GFP. The pHRSIN-GFP vector was generated by replacing the mCherry sequence and multiple cloning site (MCS) from the PHRSIN-JJ vector (a kind gift from John James at Warwick University, UK) with GFP and the MCS from pLXIN2, using XhoI and NotI restriction sites. Site-directed mutagenesis was used to generate point mutations in the pleckstrin homology (PH) domains of MYO1F (K770A and R780A) and MYO1E (K772A and R782A).

### Cell culture

RPE (hTERT RPE-1; ATCC CRL-4000) cells were grown in 1:1 DMEM/F12-HAM (Thermo Fisher Scientific, 11320074) supplemented with 10% fetal bovine serum (FBS) (Sigma-Aldrich, F7524), 100 U/ml penicillin and 100 μg/ml streptomycin (Sigma-Aldrich, P4333). U937 cells (ECACC, Salisbury, UK, 8501140) were maintained in RPMI-1640 (Sigma-Aldrich, R8758) supplemented with 10% FBS, 100 U/ml penicillin and 100 μg/ml streptomycin. THP-1 cells (ECACC, 88081201) were cultured in RPMI-1640 (Sigma-Aldrich, R8758) supplemented with 50 µM β-mercaptoethanol (Thermo Fisher Scientific, 31350010), 10% FBS, 100 U/ml penicillin and 100 μg/ml streptomycin. Differentiation of THP-1 cells into macrophage-like cells was induced by treatment with 50 ng/ml phorbol 12-myristate 13-acetate (PMA) (Sigma-Aldrich, P8139) for 24 h, followed by a 48 h recovery period in standard THP-1 medium. HEK293T (ATCC, CRL-3216) and Phoenix cells (Phoenix-AMPHO; ATCC CRL-3213) were cultured in RPMI-1640 containing 10% FBS, 100 U/ml penicillin and 100 μg/ml streptomycin. Finally, RAW246.7 macrophages (ECACC) were cultured in DMEM (Sigma-Aldrich, D5030) supplemented with 10% FBS and grown at 37°C, 10% CO_2_, and split every 2–3 days.

Stable RPE and U937 cell lines were generated using the Phoenix retroviral expression system with the pLXIN retroviral packaging vector. Virus was generated by transfection of Phoenix HEK293T cells with the pLXIN2 vector. The virus was harvested after 48 h and used to infect target cells before selection with 0.5 mg/ml G418 (Thermo Fisher Scientific, 10131027).

Transient transfections of RPE cells were performed using Fugene 6 (Promega UK Ltd, Southampton, Hampshire, UK; E2693) for immunofluorescence or PEI (Polysciences, 23966) for immunoprecipitations according to the manufacturer's instructions.

Lentiviral transduction of THP-1 cells was performed as follows: virus was generated by transfection of HEK293T cells with pHRSIN-GFP and the packaging vectors gagpol and VSVG using Lipofectamine 2000 (Thermo Fisher Scientific, 11668019). After 48 h, virus was harvested and used to infect THP-1 cells.

Knockdowns were performed by transfection of ON-TARGETplus SMARTpool siRNA oligonucleotides (Horizon Discovery, Lafayette, CO, USA) into RPE cells using Oligofectamine (Thermo Fisher Scientific, 12252011). To ensure efficient knockdown transfections were performed on days 1 and 3.

### Mouse microglia isolation

Primary microglia were isolated from mouse pups on postnatal day (P)1 to P3. Brains were extracted into Hanks' Balanced Salt Solution (HBSS). The cortex hemispheres were separated and the meningeal membranes removed. Cortices were transferred to a dish with HBSS. Tissue was minced with small scissors and incubated with 2 ml trypsin (0.05%) and DNase (7500 U, 5 mg/ml Sigma) at 37°C for 5 min before further dissociating the cell suspension using a glass pipette. 8 ml medium [DMEM (Sigma-Aldrich, D6429) with 10% heat-inactivated FBS (Sigma-Aldrich, F7524) and penicillin-streptomycin] was added to inhibit the trypsin. The suspension was then centrifuged at 1400 rpm (200 ***g***) for 5 mins and the supernatant removed. The cell pellet was resuspended in 6 ml medium and transferred to poly-D-lysine-coated (Sigma-Aldrich, P0899) T25 flasks (1 brain per T25 flask) and incubated at 37°C. Medium was changed the following day and then alternate days until a confluent layer of astrocytes with a layer of microglia on top were visible. After 7–10 days microglia were removed from the mixed glial culture by tapping the flask against a glass bottle until the microglia detached. Microglia were then replated on poly-D-lysine-coated 6-well plates containing coverslips and used for immunofluorescence. Astrocytes adherent to the flask after microglia tap-off were washed with HBSS and harvested for western blotting.

The mice were bred and housed under pathogen-free conditions in the animal facility at Cambridge University. Experimentation involving animals was carried out under a UK Home Office Project Licence granted to Dr Folma Buss (PPL 80/2372) and was approved by the UK Home Office and the University of Cambridge Animal Welfare and Ethical Review Committee. The work has been carried out in accordance to the UK Animals (Scientific Procedures) Act 1986 and follows the Laboratory Animal Science Association (LASA) Guidelines.

### Generation of human iPSC-derived microglia

Microglia were differentiated from the human iPSC line, KOLF2.1J (Jackson Laboratory, JIPSC001000) (Pantazis et al., 2022). The KOLF2.1J iPSCs were grown in StemFlex medium (Thermo Fisher Scientific, A3349401) on Geltrex-coated (Thermo Fisher Scientific, 12063569) plates and passaged with ReLSR at least twice post-thaw prior to commencing the microglial differentiation protocol. The iPSCs were cultured to 70–80% confluency and enzymatically dissociated using Accutase (STEMCELL Technologies, Waterbeach, Cambridge, UK; 07922) at 37°C for 10 min, before seeding at 10,000 cells per well of a 96-well Clear Round Bottom Ultra-Low Attachment Microplate (Corning, 7007) in mTeSR medium (STEMCELL Technologies, 100-0276) containing 10 μM ROCKi (Sigma-Aldrich, SCM075), 50 ng/ml BMP4 (PeproTech, NJ, USA; 120-05), 50 ng/ml VEGF (PeproTech, 100-20) and 50 ng/ml SCF (PeproTech, 300-07). Treatment with BMP4 induced mesoderm, VEGF endothelial precursors and SCF hematopoietic precursors, resulting in the formation of embryoid bodies (EBs).

From day 2, EBs were cultured in medium without ROCKi, and on day 4, ∼30 EBs were transferred into a T25 cell culture flask containing macrophage-directed differentiation medium consisting of X-vivo 15 (Lonza, BE02-060F) supplemented with 100 ng/ml M-CSF (Thermo Fisher Scientific, PHC9504), 25 ng/ml IL-3 (Thermo Fisher Scientific, PHC0035), 2 mM GlutaMAX, 0.05 mM β-mercaptoethanol (Thermo Fisher Scientific, 31350010) and 1× Antibiotic-Antimycotic (Thermo Fisher Scientific, 15240062). After a week, a complete medium change was performed, thereafter only two-thirds of the medium was changed every 4–5 days. The EBs attached to the plate, were surrounded by adherent stromal cells and formed cystic structures reminiscent of yolk sac morphology.

Macrophage precursors emerged in the culture supernatant after 2–3 weeks and were collected at 4–5 day intervals during weeks 3 to 5 of differentiation. Macrophage precursors were seeded onto coverslips at a density of 200,000 cells per well of a 12-well plate. To promote the maturation of macrophage precursors into a microglial phenotype, cells were cultured for 14 days in a differentiation medium composed of DMEM/F12 supplemented with 1× N2 (Thermo Fisher Scientific, 17502038), 2 mM GlutaMAX (Thermo Fisher Scientific, 35050038), 50 μM β-mercaptoethanol, 100 ng/ml interleukin-34 (PeproTech, 200-34), 10 ng/ml granulocyte-macrophage colony-stimulating factor (Thermo Fisher Scientific, 2907249) and 50 U/ml Antibiotic-Antimycotic. The cultures were replenished with fresh medium every 4–5 days. Macrophages matured into microglia after 14 days in culture.

### Immunofluorescence microscopy

Cells were grown on coverslips, washed with 1× PBS and fixed in 4% paraformaldehyde for 20 min. After washing with PBS, cells were permeabilised for 5 min with 0.2% Triton X-100 and blocked for 1 h with 1% BSA in PBS before incubating with the indicated primary antibodies for at least 1 h at room temperature or at 4°C overnight. Primary antibodies were detected by incubating for 1 h with AlexaFluor488- or AlexaFluor568-coupled secondary antibodies (Thermo Fisher Scientific). F-actin was visualised using phalloidin coupled to either AlexaFluor568 or AlexaFluor647 (Thermo Fisher Scientific, A12380, A22287), and cell nuclei were stained with Hoechst 33342 (Thermo Fisher Scientific). Cells were mounted on slides using ProLong Antifade reagent (Thermo Fisher Scientific, P36936), imaged using the Zeiss SP880 confocal microscope with or without Airyscan using a 100× objective. Images were processed using FIJI (https://fiji.sc/).

### Immunoprecipitation and western blotting

Immunoprecipitations were performed from RPE wild-type or stable cell lines. At 24 h after transfection, cells were lysed in 50 mM Tris-HCl pH 7.4, 100 mM NaCl, 1% NP-40, 5 mM MgCl_2_, 5 mM ATP and complete protease inhibitor cocktail (Roche). After shearing with a 25-gauge needle, lysates were clarified by centrifugation at 20,000 ***g*** for 15 min at 4°C. Supernatants were precleared with protein A Sepharose (Cytiva) followed by incubation with 5 µg antibody for 1 h, then protein A Sepharose for 1 h. After washing the beads three times with lysis buffer and once with PBS, proteins were boiled in SDS loading buffer and separated by SDS-PAGE. After protein transfer on to nitrocellulose (Protran, Amersham), the membrane was blocked with 5% milk in PBS-T (0.05% Tween-20 in PBS), incubated with primary antibodies overnight at 4°C, washed with PBS-T and incubated with the corresponding HRP-conjugated secondary antibody (Sigma) for 1 h at room temperature. After washing, bound antibody was detected using enhanced chemiluminescence (ECL) substrate (GE Healthcare Life Sciences) according to the manufacturer's protocol and exposed to X-ray film (Fujifilm) or imaged using a G:BOX imaging system (Syngene). Raw blots are provided as Fig. S9.

### BioID purification and sample processing

RPE cells (3×150 mm dishes at 50% confluency) or U937 cells (∼4×10^7^ cells) were supplemented with 50 µM biotin in growth medium and incubated for 24 h. Cells were lysed with RIPA lysis buffer [50 mM Tris-HCl pH 7.4, 150 mM NaCl, 1% NP-40, 0.5% sodium deoxycholate, 1 mM EDTA, 0.1% SDS and complete protease inhibitor cocktail (Roche cOmplete Mini, EDTA-free, 11836170001)], sonicated and centrifuged at 20,000 ***g*** for 15 min at 4°C, and the supernatants were mixed with high-capacity streptavidin beads (Thermo Fisher Scientific, 20357) for 3 h at 4°C. Beads were washed with RIPA buffer, TBS and 50 mM ammonium bicarbonate pH 8 before incubation with 10 mM dithiothreitol (DTT) for 30 min at 56°C. The solution was spiked with 10 µl 550 mM iodoacetamide (Sigma-Aldrich BioUltra, I1149) for 20 min at room temperature, and the beads washed in ammonium bicarbonate before digestion overnight with 0.5 µg Trypsin Gold (Promega, V5280) at 37°C. An additional 0.5 µg trypsin was added the following day and incubated for a further 2 h. After pelleting the beads, the supernatants were collected and the beads washed twice with 150 µl HPLC-grade H_2_O (Sigma-Aldrich, CHROMASOLV) and all supernatants combined. These were spiked with 1 µl trifluoroacetic acid (TFA) and dried to a pellet in a vacuum centrifuge.

### Mass spectroscopy acquisition and data analysis

Samples were resuspended in mass spectrometry (MS) solvent (3% acetonitrile, 0.1% TFA) for analysis on a Q Exactive Plus (Thermo Fisher Scientific) coupled to an RSLC3000nano UPLC (Thermo Fisher Scientific). Peptides were resolved using a 50 cm C18 PepMap EASYspray column with a gradient rising from 97% solvent A (0.1% formic acid), 3% solvent B (80% acetonitrile, 0.1% formic acid) to 40% solvent B over 40 min. Data were acquired in a top ten data-dependent acquisition fashion, with MS spectra acquired between *m/z* 400 and 1500 at 70,000 full width at half maximum (fwhm). MS-MS spectra were acquired at 17,500 fwhm and excluded from further fragmentation for 30 s. Raw files were processed as a single batch using the MaxQuant proteomics software package version 2.4.13.0 (Cox and Mann, 2008). Spectra were searched against a reviewed Uniprot *Homo sapiens* database (downloaded 24/10/23). Cysteine carbamidomethylation was set as a fixed modification, and methionine oxidation and N-terminal acetylation were selected as variable modifications. Both peptide and protein false discovery rates (FDRs) were set to 0.01, the minimum peptide length was set at eight amino acids, and up to two missed cleavages were tolerated.

Bioinformatics analysis was performed in the Perseus package bundled with MaxQuant (Tyanova et al., 2016). Data were filtered by removing matches to the reverse database, proteins only identified with modified peptides and common contaminants, and intensity values were log_10_ transformed.

Volcano plots were generated using Perseus v. 2.0.11. LFQ intensities were filtered for reverse database matches, potential contaminants, and proteins only identified by modification site, and then transformed by log_2_(x). Proteins were filtered based on a minimum of three valid values, and missing values were imputed with a width of 0.3 and downshift of 2.0. The false discovery rate was set at 0.01 and the S0 value at 2.0.

### Phagocytosis assay and image acquisition

RAW246.7 cells (∼400,000 cells) were nucleofected with the Lonza Nucleofector system (SF Cell Line, Lonza). Cells were pelleted by centrifugation at 90 ***g*** for 5 min and resuspended in 20 µl of Nucleofector solution supplemented with 0.4 µg of plasmid DNA. Following nucleofection, cells were incubated for 5 min at room temperature before being transferred to pre-warmed complete culture medium and seeded onto ethanol-sterilised glass coverslips for overnight culture.

BSA-coated polystyrene beads (6–8 µm, Spherotech, BP-60-5) were washed three times with PBS and incubated overnight at 4°C with 10 µg/ml AlexaFluor647-conjugated anti-BSA antibody (labelling kit: Thermo Fisher Scientific, A88068; antibody: rabbit polyclonal anti-mouse BSA, MP Biomedicals, Irvine, CA, USA, 08651111). After 24 h, the beads were washed three times with 1× PBS and resuspended in pre-warmed culture medium.

To initiate phagocytosis, the cell medium was replaced with the prepared bead solution (10× excess over number of cells seeded) and incubated at 37°C, 5% CO_2_ for 15 min before fixing in 4% paraformaldehyde followed by three washes with PBS.

The cells were imaged on a spinning-disc confocal microscope (Revolution; Andor) comprising a DMi8 (Leica) housing equipped with a spinning-disc unit (CSU-X1; Yokogawa), using a 60× objective (1.3NA; Leica) and 405, 488 and 561 nm lasers. Image stacks were acquired as averages of 2 at a *z*-interval of 0.2 µm with an iXon Ultra 888 EmCCD camera (Andor) set to 250 gain and 50 ms exposure.

### Image analysis

ImageJ (NIH, Bethesda, MD, USA) was used to calculate maximum-intensity *z* projections of the image stacks. To quantify the accumulation of protein intensity at the phagocytic cup, masks for the three channels (bead, actin and the protein of interest) were created via the OTSU algorithm. For the actin mask, the threshold was set at twice the calculated Otsu threshold. To segment the area of the actin that forms the phagocytic cup, the bead mask and the actin mask were multiplied to select only actin structures that did contain a fluorescent bead (see Fig. S8). The accumulation of the protein of interest was then quantified as the ratio of average protein intensity inside the actin ring (*I*_inside_) divided by the average protein intensity in the protein mask without the actin ring (*I*_outside_), minus one:

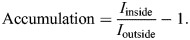
For the statistical analysis two-sided Student's *t*-tests were performed to gather the reported *P*-values (Prism 10.1.2, Graphpad).

## Supplementary Material

10.1242/joces.264357_sup1 Supplementary information
