# Supplementary material for: The MYO1F interactome reveals ASAP1, CD2AP and SH3KBP1 as novel adaptor proteins in podosomes and phagosomes
Source: J Cell Sci. 2025 Dec 22;138(24):jcs264357. doi: 10.1242/jcs.264357 (PMC12772958; doi:10.1242/jcs.264357)
Supplement: Supplementary information [file joces-138-264357-s1.pdf]

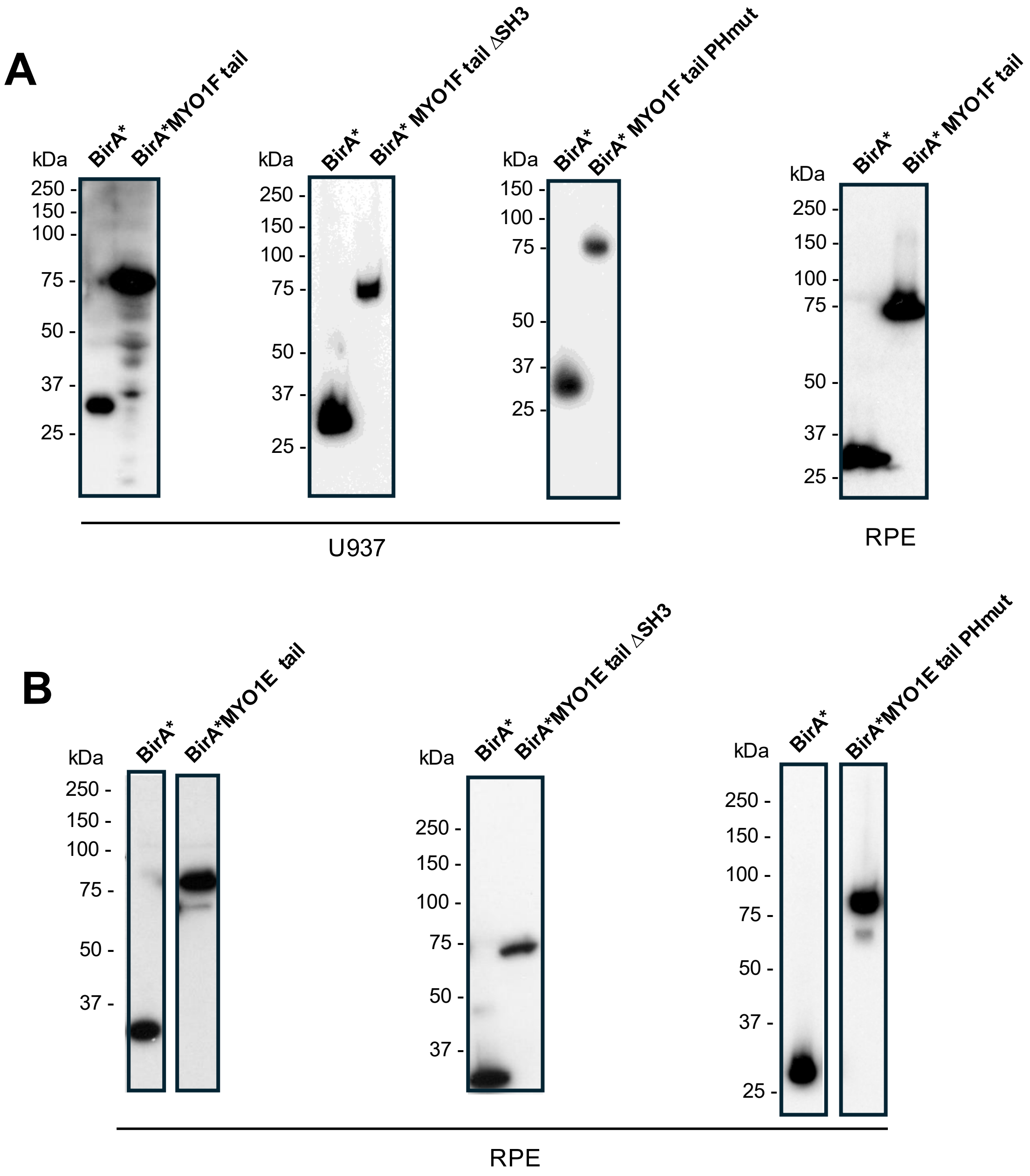

Fig. S1. Verification of BirA-MYO1F-tail or E-tail expression by immunoblotting. (A) Analysis of myc-BirA*MYO1F tail, myc-BirA*MYO1F tailΔSH3 or myc-BirA*MYO1F tail PHmut expression in U937 and RPE cells by immunoblotting using a myc antibody. **(B)** Expression of myc-BirA*MYO1E tail, myc-BirA*MYO1E tailΔSH3 or myc-BirA*MYO1E tail PHmut tested by immunoblotting using a myc antibody.

Journal of Cell Science • Supplementary information

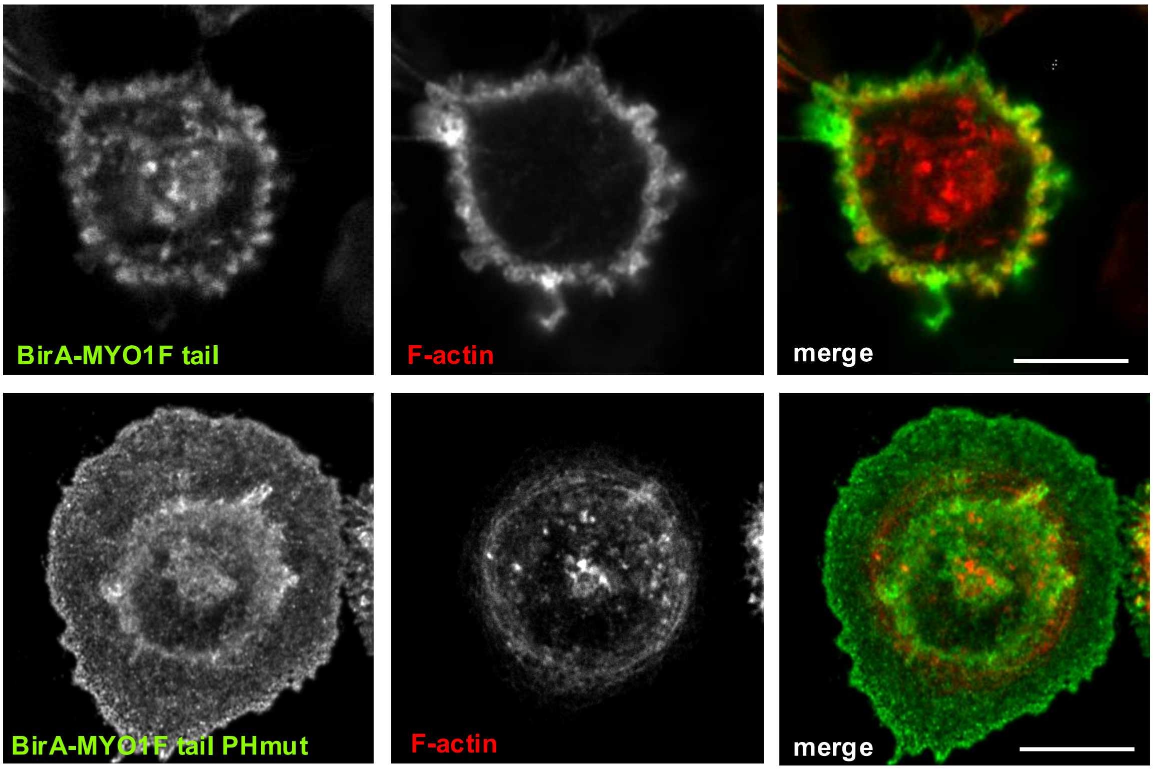

Fig. S2. Localisation of BirA-MYO1F tail and BirA-MYO1F tail PHmut in U937 cells. A. Validation of U937 cells stably expressing myc-BirA*MYO1F tail or myc-BirA*MYO1F tail PHmut by immunofluorescence using a myc antibody. Confocal images were taken at the dorsal layer of the cell close to the coverslip. Scale bar 10 µm.

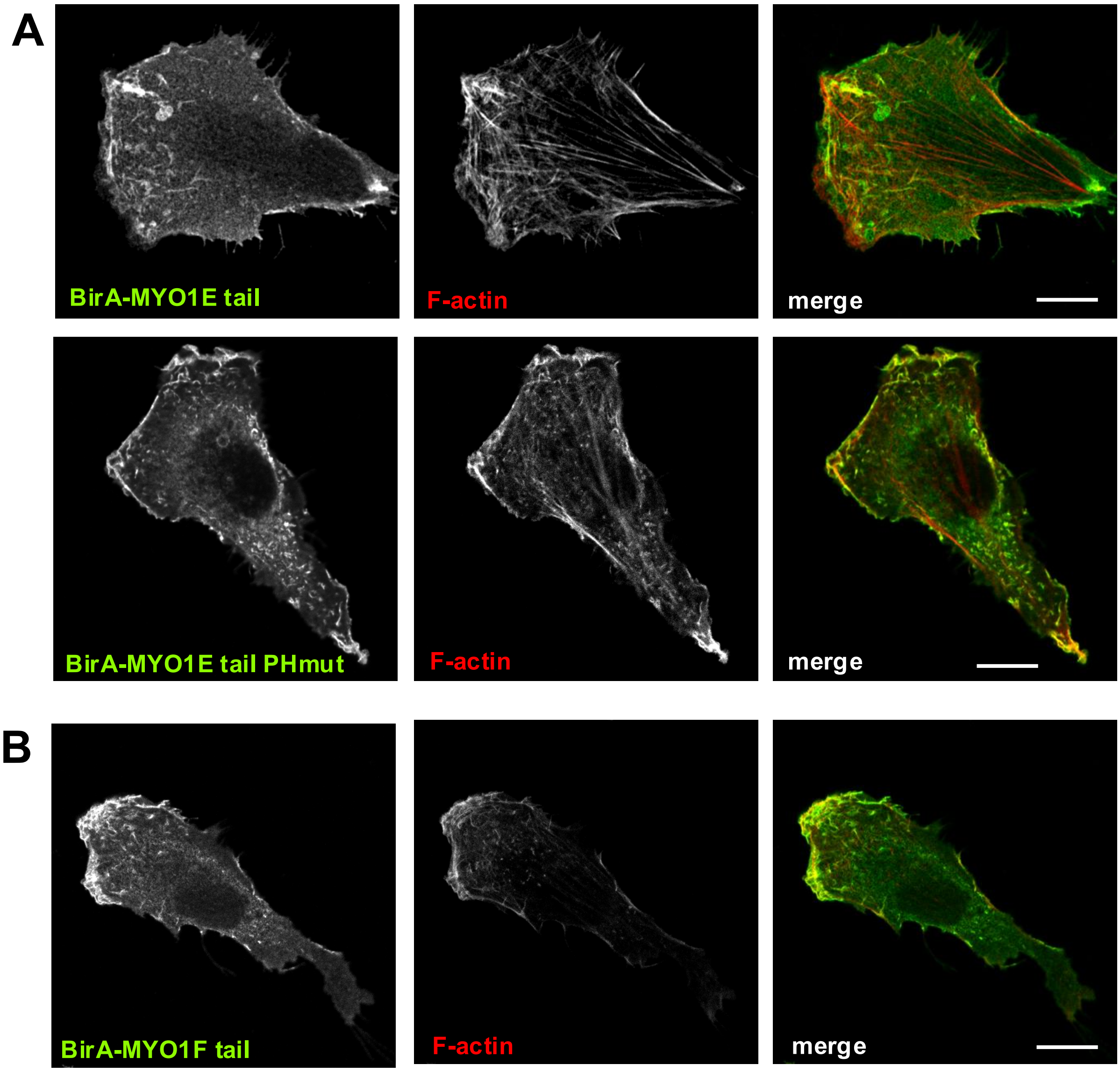

Fig. S3. Localisation of BirA-MYO1F or E in RPE cells. A. Analysis of U937 and RPE cells stably expressing myc-BirA*MYO1F tail, myc-BirA*MYO1F tailDSH3 or myc-BirA*MYO1F tail PHmut or **B**. myc-BirA*MYO1E tail, myc-BirA*MYO1E tailΔSH3 or myc-BirA*MYO1E tail PHmut by immunofluorescence using a myc antibody. Confocal images were taken at the dorsal layer of the cell close to the coverslip. Scale bar 10 µm.

Journal of Cell Science • Supplementary information

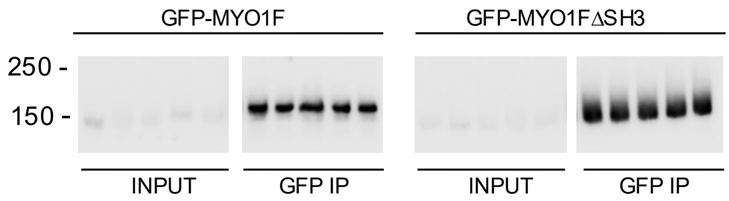

Fig. S4. GFP-MYO1F and GFP-MYO1FΔSH3 expression in figure 3 B. Shown is the expression of GFP-MYO1F and GFP-MYO1FΔSH3 in the input lane and after immunoprecipitation using antibodies to GFP.

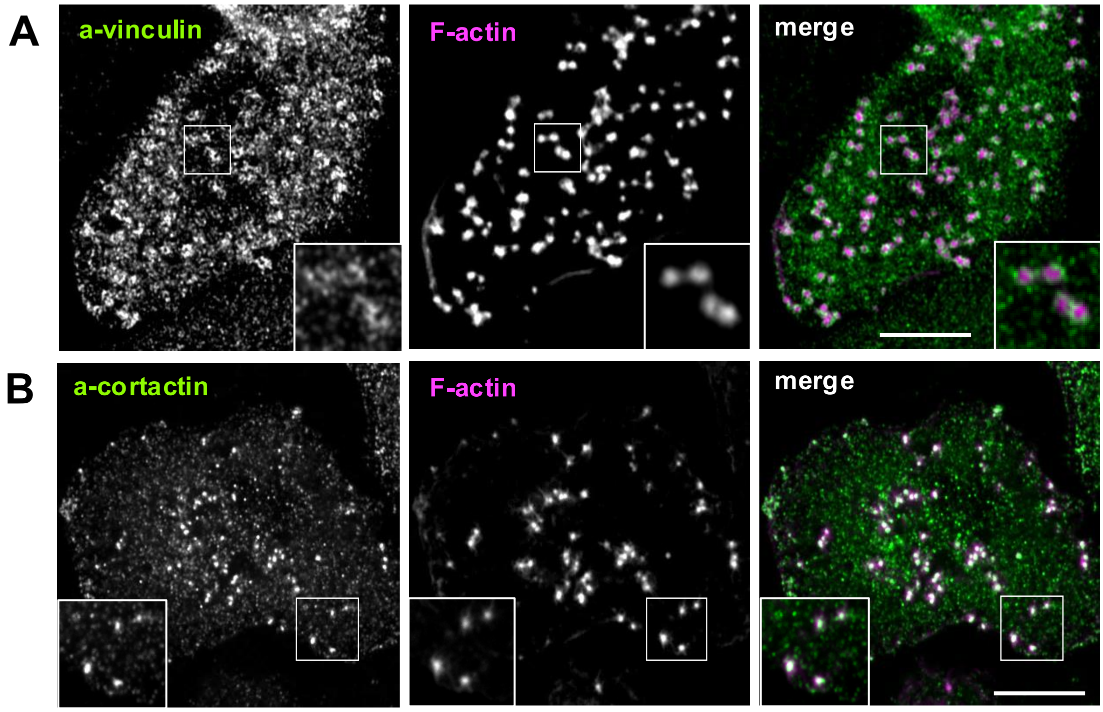

Fig. S5. The podosome marker proteins vinculin and cortactin are present in podosomes in THP-1 cells. THP-1 cells treated with PMA to induce differentiation into adherent macrophage-like cells were stained in immunofluorescence with antibodies to vinculin (A) or cortactin (B) and double labelled with fluorescently labelled phalloidin to visualise actin filaments. In the merge image on the righthand panel actin is labelled in magenta and vinculin or cortactin in green. White boxes in **A** and **B** indicate areas enlarged in the picture inset. Confocal images were taken at the dorsal layer of the cell close to the coverslip. Scale bar 10 µm.

Journal of Cell Science • Supplementary information

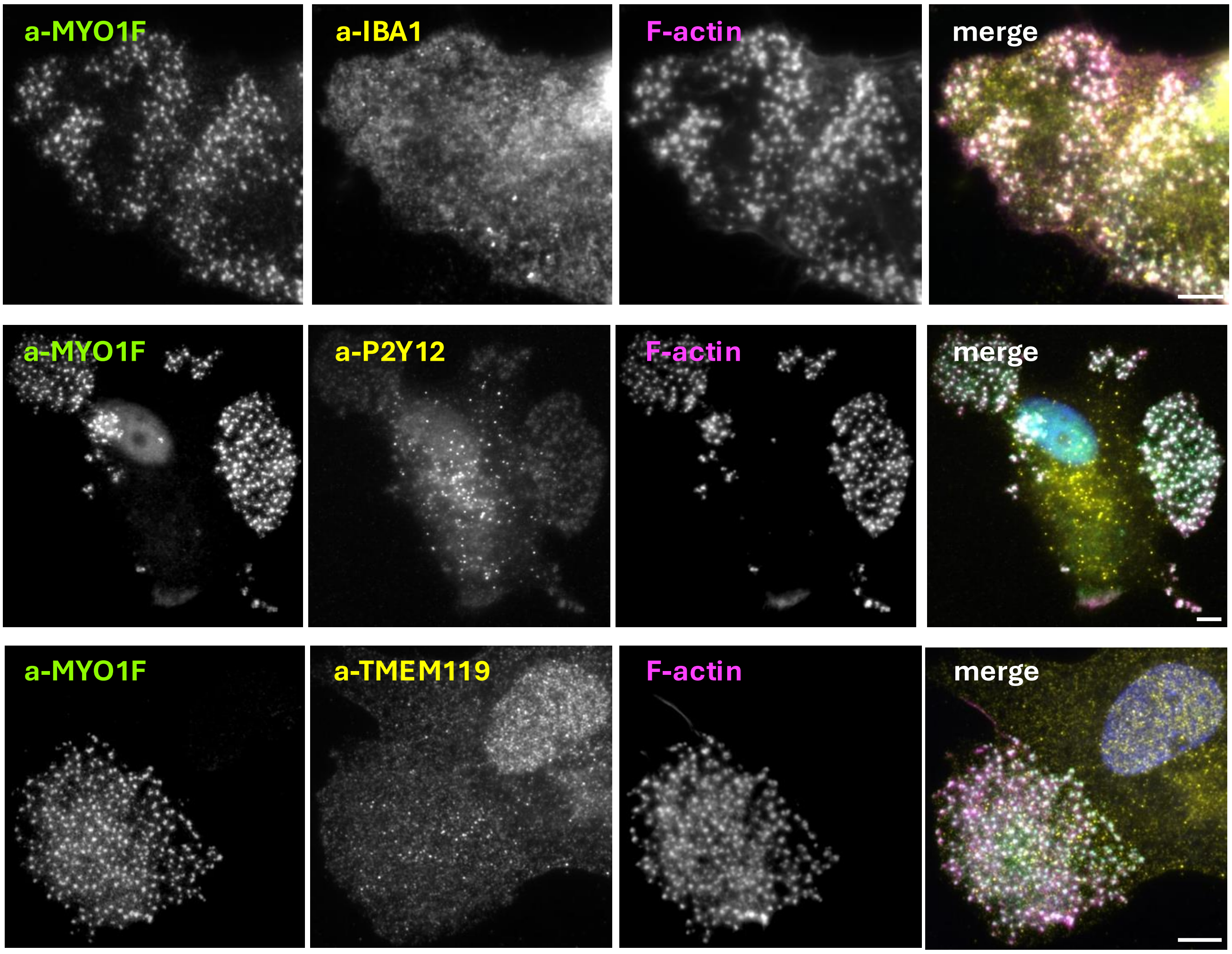

Fig. S6. Microglia marker proteins IBA1, P2Y12 and TMEM119 are expressed in iPSC-derived human microglia. Human hiPSC-derived microglia were stained with antibodies to MYO1F and IBA1, P2Y12 or TMEM119 and fluorescently-labelled phalloidin. The merge image on the righthand panel shows actin in magenta, MYO1F in green, IBA1, P2Y12 or TMEM119 in yellow and nucleus in blue. Widefield images were taken at the dorsal layer of the cell close to the coverslip. Scale bar 5 µm.

Journal of Cell Science • Supplementary information

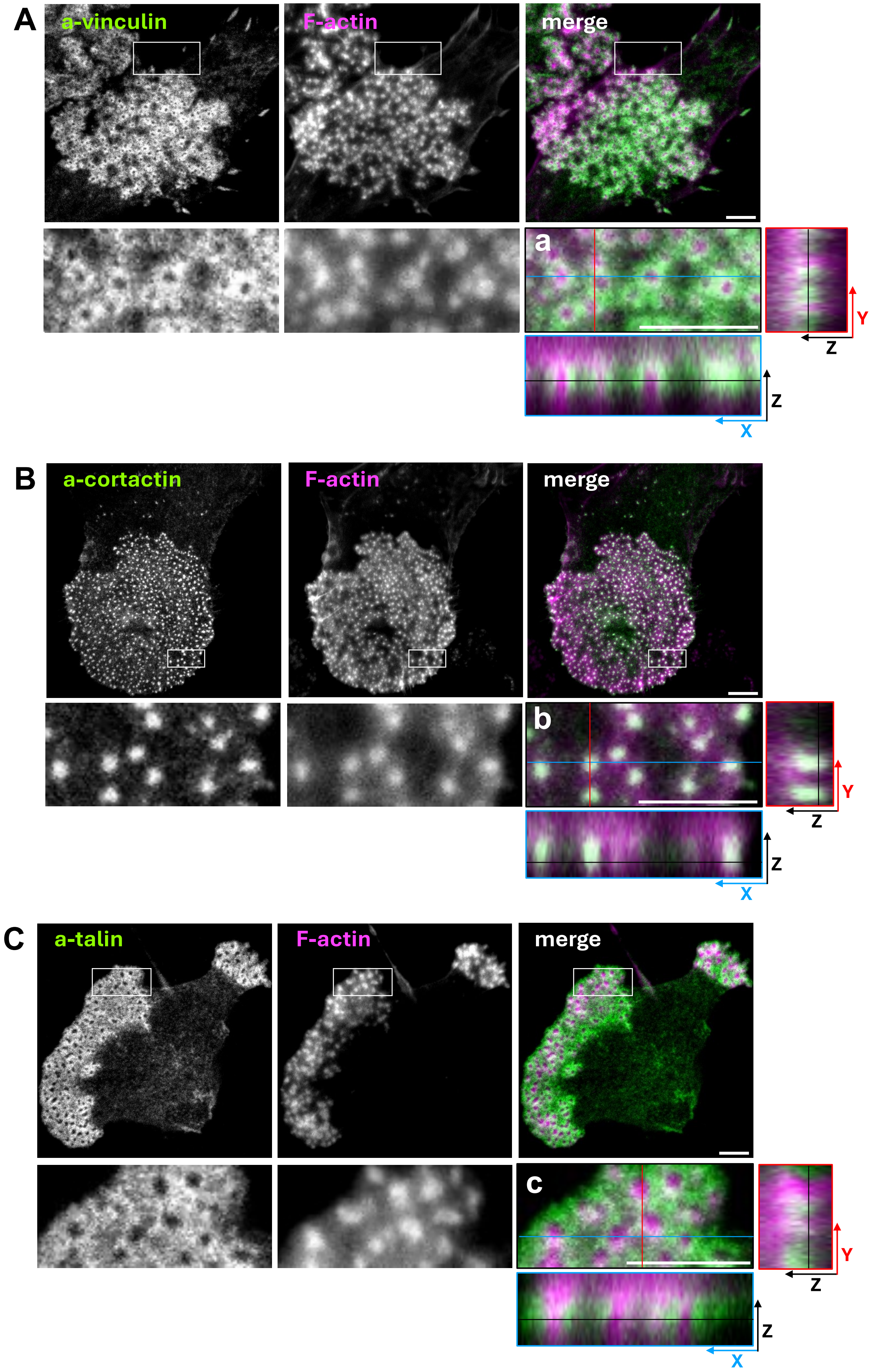

Journal of Cell Science • Supplementary information

Fig. S7. Localisation of podosome marker proteins vinculin, cortactin and talin in iPSC-derived human microglia . Human iPSC-derived microglia were stained with antibodies to vinculin (**A**), cortactin (**B**) or talin (**C**) and double labelled with fluorescently-conjugated phalloidin to visualise actin filaments. In the merge image on the righthand panel actin is shown in magenta and vinculin, cortactin or talin in green. Confocal z-stacks were taken at the dorsal layer of the cell along the hight of podosomes. White boxes in **A, B** and **C** indicate areas enlarged in the picture below. Images below and to the right of the enlarged merged XY views in A. B and C show the XZ view along the blue line and YZ view along the red line, respectively. Scale bar 5 µm.

Journal of Cell Science • Supplementary information

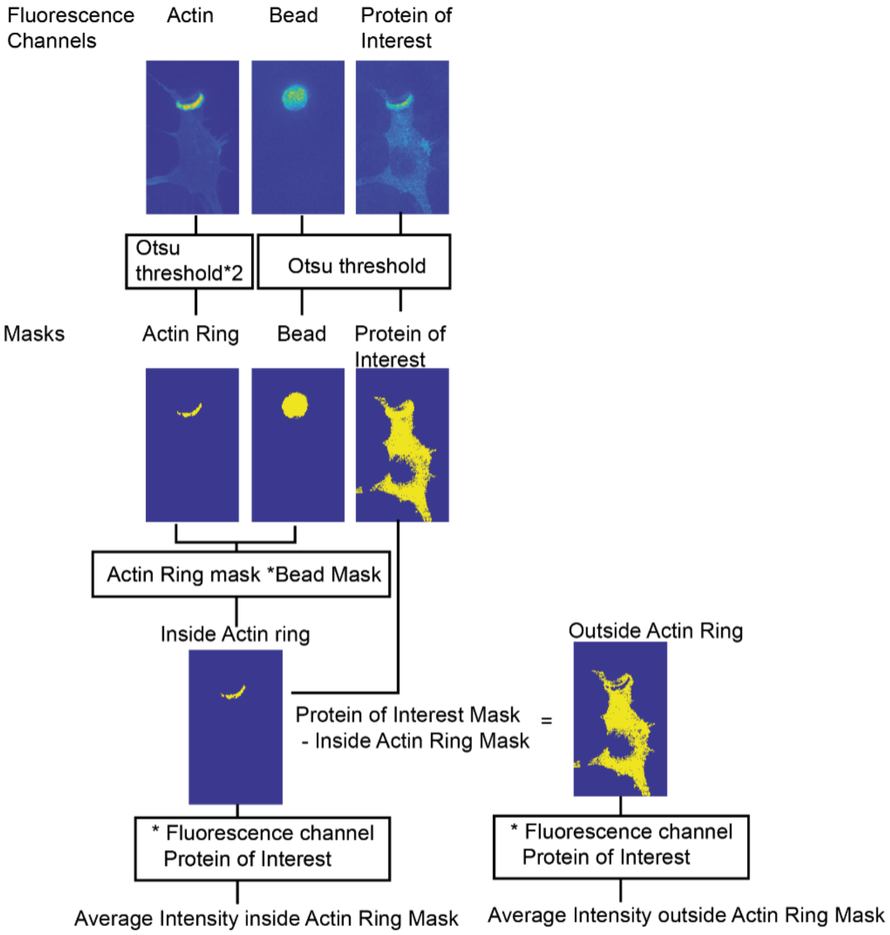

$$\text{Accumulation} = \frac{\text{Average Int. inside Actin Ring}}{\text{Average Int. outside Actin Ring}} - 1$$

Fig. S8. Image analysis of phagocytosis assay. Each fluorescence channel was first normalised to the maximum pixel intensity. Otsu thresholding was then calculated for each fluorescence channel to generate the protein of interest mask as well as the bead mask. To identify areas of high actin accumulation (the actin ring), the Otsu threshold for the actin channel was multiplied by two to generate a high actin mask. To compare the intensity of the protein of interest inside versus outside of the actin ring, two additional masks were generated, an inside actin mask and an outside actin mask. The inside actin mask was generated by multiplying the bead mask with the actin ring mask to ensure that only high actin structures engulfing a bead were included. The outside actin mask was created by subtracting the inside actin mask from the protein of interest mask. The average intensity of the protein of interest was then compared using an accumulation value which divided the average intensity of the protein of interest inside the actin ring by the average intensity outside the actin ring. The resulting value was then subtracted by one.

Journal of Cell Science • Supplementary information

Table S1. MYO1F BioID Data. Gene IDs, difference over control and p-value scores are shown

Myo1F-control			MYO1F ΔSH3-control			MYO1F ΔPH-control		
Gene name	Difference	-Log p value	Gene name	Difference	-Log p value	Gene name	Difference	-Log p value
MYO1F	9.52	16.81	MYO1F	8.43	11.77	MYO1F	10.20	12.72
PHACTR4	7.01	9.76	ACTB	7.43	1.74	PHACTR4	6.07	6.03
PLEKHO2	6.96	15.69	SPTBN1	7.03	3.57	PRRC2A	5.96	9.54
ERBB2IP	6.27	9.89	ACTC1;ACTA	6.90	1.27	NAP1L4	5.78	3.03
ASAP1	6.23	10.66	SPTAN1	6.90	3.22	CSDE1	5.74	3.14
DOCK8	6.08	9.42	IQGAP1	6.44	2.48	ASAP1	5.46	7.06
ZDHHC5	5.83	10.69	WDR1	6.42	4.67	NAP1L1	5.44	2.71
SH3BP2	5.75	9.89	ACTN4	6.26	1.56	YTHDF2	5.43	6.52
ESYT1	5.59	6.63	ERBB2IP	6.18	8.27	NUFIP2	5.29	3.27
DLG1	5.43	10.10	FAM129B	6.12	9.93	XRN1	5.28	3.03
NUMB	5.29	10.22	ACTN1	5.94	2.02	SH3BP2	5.20	6.80
MAP4K4	5.27	8.63	RASA3	5.65	8.82	FAM120A	5.18	6.69
UTRN	5.25	6.26	CORO1C	5.60	2.82	IPO7	5.16	9.62
FAM129B	5.17	10.13	AIF1	5.59	3.14	PLEKHO2	5.12	9.90
LAT2	5.15	5.62	TMOD3	5.57	2.40	PCM1	5.09	2.76
ESYT2	5.12	6.89	LIMA1	5.57	3.15	PRRC2C	5.07	4.92
SH3KBP1	5.11	4.65	ZDHHC5	5.22	8.59	UBAP2	5.03	4.29
PPFIBP2	5.10	11.83	DLG1	5.17	7.32	UBAP2L	4.97	2.57
SLC4A7	5.01	6.18	MYO1G	5.12	2.40	ZDHHC5	4.96	8.27
RASA3	4.85	7.34	PVRL2	5.12	6.50	TDRD3	4.90	7.37
CD99	4.82	3.85	TWF2	5.11	2.05	LCP2	4.89	3.87
STEAP3	4.82	4.90	CAPZA2	5.10	1.77	DVL1;DVL1P	4.82	4.11
MARK3	4.79	10.54	ITGA6	5.05	3.07	ATXN2L	4.73	2.13
SLC30A1	4.65	6.37	PHACTR4	5.03	5.05	UBE2O	4.73	3.43
SIRPA;SIRPB:	4.63	6.05	SLC38A1	5.00	7.61	LARP1	4.70	2.02
AHCYL1	4.61	6.52	ACTG1	4.99	2.15	ANKRD17	4.70	4.51
GPR124	4.60	9.40	PVRL1	4.98	8.27	STRAP	4.65	2.75
CD2AP	4.56	4.05	ESYT1	4.98	3.91	MAP7D3	4.65	6.55
WAS	4.54	3.69	CFL1	4.95	2.19	YTHDF3	4.63	9.06
SLC7A5	4.52	3.87	DOCK8	4.88	5.71	WAS	4.60	2.33
EFR3A	4.42	7.79	CD44	4.86	3.40	MAP4K5	4.59	6.81
BASP1	4.37	8.81	MAP4K4	4.83	5.68	EIF3L	4.57	3.75
ADD3	4.36	10.11	RELL1	4.83	7.29	UPF1	4.57	5.19
MARK2	4.29	7.22	TWF1	4.81	3.22	MAP4K4	4.47	5.30
NF2	4.28	8.74	LAT2	4.75	3.31	SND1	4.46	2.21
GOLGA8R	4.27	2.45	NDRG1	4.66	2.58	LSM12	4.46	2.66
CD44	4.26	3.26	EFR3A	4.65	6.48	ATXN2	4.45	6.65
CDCA3	4.19	12.74	MARK3	4.57	7.87	ZC3HAV1	4.44	2.83
ITGA6	4.15	3.78	BASP1	4.50	6.76	EIF4B	4.43	1.63
FNBP1	4.12	6.85	CD99	4.47	2.18	ZCCHC6	4.43	7.52
SNAP29	4.10	4.56	MCEMP1	4.38	4.87	ERBB2IP	4.41	6.31
CDC37	4.08	4.12	CAPZB	4.36	1.99	SERBP1	4.37	2.74
FYB	4.07	4.69	ESYT2	4.36	3.91	GEMIN5	4.30	1.57
KANK2	4.06	3.37	NF2	4.35	6.76	NACA	4.29	2.16
SH3GL1	4.06	5.12	CORO2B	4.31	4.03	EIF4G1	4.28	1.74
SIGLEC6	4.04	3.90	LRRC25	4.29	7.90	SH3KBP1	4.23	2.31
PECAM1	4.02	9.05	SIRPA;SIRPB	4.28	5.46	GIGYF2	4.22	2.36
MARCKS	4.01	6.48	FCER1G	4.27	7.24	EIF3B	4.17	2.45
NDRG1	3.97	3.39	ACTBL2	4.26	2.00	EIF2A	4.12	1.93
SNAP23	3.96	6.01	UTRN	4.25	3.30	DVL3	4.12	2.58
SIGLEC5;SIGI	3.95	6.36	KANK2	4.25	2.21	CD2AP	4.10	2.20
EPB41	3.89	8.62	AHCYL1	4.12	5.07	SIGLEC6	4.10	2.91
PACSIN2	3.88	4.85	NUMB	4.10	7.29	EIF3E	4.09	3.11
REPS1	3.87	4.21	SNAP23	4.07	4.36	TCHP	4.05	2.51
MAP4K5	3.85	5.09	CORO2A	4.07	4.50	ZC3H15	4.00	2.15
LIMD1	3.84	4.07	ARPC3	4.02	1.77	ESYT1	3.99	3.04
RELL1	3.77	3.77	CAPZA1	3.89	2.27	EIF3G	3.96	2.47
SLC3A2	3.71	3.22	PECAM1	3.88	7.47	EIF4G2	3.95	3.12
SNX9	3.68	9.00	ARPC2	3.86	1.65	ZYX	3.91	1.39
WDR44	3.66	6.48	ARPC5	3.85	1.47	PRAM1	3.84	1.94
HMHA1	3.65	6.09	SLC4A7	3.85	2.96	BRAP	3.83	2.08
PRAM1	3.62	2.76	CPNE8	3.83	7.33	DHX29	3.81	4.35
TBC1D10B	3.62	8.20	TMOD2	3.81	3.78	CRKL	3.80	1.31
JAG1	3.61	10.87	FRMD3	3.77	6.89	G3BP1	3.77	3.59
ZC3HAV1	3.60	3.41	SLC30A1	3.74	3.96	EML4	3.73	4.91
PVRL2	3.60	4.00	GPR124	3.70	7.04	DDX6	3.71	2.75
GAB1	3.52	9.90	CD33	3.68	6.42	LIMD1	3.70	2.87
PEAK1	3.45	5.93	ARPC1B	3.66	1.69	PACSIN2	3.69	7.07
SBF1	3.39	5.93	SIGLEC6	3.66	2.53	RGL2	3.68	4.92
PPFIBP1	3.32	8.23	SNAP29	3.61	2.66	YTHDF1	3.68	5.28
WIPF1	3.30	4.29	RAB11A;RAE	3.61	4.78	UTRN	3.67	2.77
FMNL1	3.30	3.88	RASA2	3.56	6.51	MAPRE2	3.66	1.62
SEMA4D	3.29	6.21	ITGA5	3.56	2.45	RALBP1	3.66	2.98
PVRL1	3.28	3.47	MYL6	3.51	1.75	SLC7A5	3.65	2.23
SLC39A10	3.28	3.00	JAG1	3.49	7.44	R3HDM1	3.64	6.14
ADD1	3.28	6.77	SLC9A3R1	3.48	2.14	EIF4E2	3.62	3.68
EPB41L3	3.27	8.42	HLA-A	3.48	2.21	SKA3	3.62	2.48
NCKAP1L	3.19	4.32	SLC39A10	3.46	4.78	FAM129B	3.61	6.67
HLA-A	3.19	3.18	MARK2	3.46	5.33	SYNJ2	3.61	4.90
EHBP1	3.18	5.79	PPFIBP2	3.43	6.03	SLC38A1	3.59	5.79
EPS15L1	3.17	1.94	ADD3	3.40	8.90	LUZP1	3.59	2.08
ARHGAP30	3.16	4.64	EPB41L3	3.35	5.82	HAUS6	3.58	2.98
DENND3	3.16	3.27	MTMR1	3.34	4.54	PGAM5	3.57	1.58
STK10	3.15	4.25	EPB41	3.27	5.05	FYB	3.57	3.02
RASAL3	3.12	3.85	TBC1D10B	3.24	5.86	ANKRD28	3.57	3.44
SPTBN1	3.11	1.91	STEAP3	3.23	1.96	EIF3K	3.56	2.43
CYFIP1	3.10	3.68	LAIR1	3.22	2.68	EIF2S2	3.55	3.45
ITGA4	3.09	3.14	PPFIBP1	3.17	5.32	EIF3I	3.55	2.17
SASH3	3.09	4.18	SLC16A3	3.16	3.57	WIPF1	3.54	4.17
MTMR1	3.08	4.83	PAG1	3.15	5.15	CD44	3.53	2.35
ITGB1	3.07	2.60	MARCKS	3.15	3.40	EIF3C;EIF3CL	3.52	2.37
FLVCR1	3.06	5.76	CLDND1	3.13	5.81	EPS15L1	3.52	1.43
BTK	3.03	2.92	PLEKHO2	3.12	6.49	AHCYL1	3.51	4.30
SEMA4C	2.99	3.76	KDELR1	3.10	3.46	TOP3B	3.49	9.09

PSD4	2.98	3.68
CD300LF	2.96	3.63
ELMO1	2.96	4.01
YWHAG	2.94	3.64
EVI2B	2.92	2.96
ATP2B4	2.92	3.64
ITGA5	2.91	2.81
SLC38A1	2.90	2.55
ATP8B4	2.88	3.06
PTPRA	2.88	2.98
CLDND1	2.86	2.90
SIGLEC12	2.84	2.60
ROCK1	2.81	3.67
RALBP1	2.79	2.42
MPP7	2.77	3.63
SLC16A3	2.72	3.76
FCER1G	2.70	2.58
BIN2	2.68	3.33
LAIR1	2.66	2.45
SLC19A1	2.62	2.91
LRCH1	2.58	3.70
YWHAE	2.57	2.63
LRRC25	2.56	2.77
DEPDC1B	2.56	3.79
RAB11A;RAB	2.53	2.42
SDK1	2.52	4.20
PPFIA1	2.51	4.85
RFTN1	2.45	2.75
EFNB1	2.38	3.13
CPNE8	2.36	3.13
PTPN22	2.33	3.25
PTPRC	2.30	4.12
ROCK2	2.28	5.70
GOLGA8N;G	2.27	3.33
AHNAK	2.26	7.31

ITGA4	3.03	2.95
SNX9	3.03	6.02
KCNN4	3.01	6.08
SIGLEC5;SIG	2.98	3.92
RAP2C;RAP2	2.59	7.60

DVL2	3.49	1.69
ESYT2	3.49	3.03
LRRC25	3.49	6.80
MAPRE1	3.48	1.86
LAT2	3.47	2.26
SH3GL1	3.45	2.92
MAP4	3.44	1.48
PPFIBP2	3.43	6.32
HAUS4	3.40	5.42
REPS1	3.38	2.49
CBL	3.38	2.84
HDLBP	3.36	3.48
DOCK8	3.36	3.96
DLG1	3.35	4.85
EIF4G3	3.35	3.10
SNAP29	3.34	2.41
HAUS5	3.33	4.19
HAUS7	3.33	3.77
EIF4E	3.32	2.13
ASCC3	3.32	3.07
STAU2	3.31	5.54
EVI2B	3.31	5.44
CDC37	3.28	1.93
SKAP2	3.28	3.38
STEAP3	3.24	2.00
USP10	3.23	2.99
PIK3AP1	3.23	7.18
EIF3A	3.23	2.45
HAUS3	3.21	4.24
DRG1	3.21	2.28
EIF3M	3.20	2.64
KIAA0430	3.20	6.54
BTF3	3.19	2.39
LSM14A	3.18	5.05
SIGLEC5;SIG	3.18	4.22
EIF5B	3.17	2.18
FMR1	3.13	6.65
PVRL1	3.12	5.71
CNOT1	3.10	4.44
DENND3	3.07	2.27
ELMO1	3.07	4.18
PLK1	3.06	4.66
CYFIP1	3.06	2.79
EIF3F	3.05	2.02
DDX20	3.02	3.23
PPP6R1	3.02	2.79
TRIM25	3.01	2.68
ARHGAP30	2.98	5.81
OFD1	2.97	4.41
FARSA	2.91	2.27
LSG1	2.89	4.85
AP2B1	2.87	3.61
DHX57	2.87	6.17
EFR3A	2.85	4.03
MTDH	2.82	2.47
KIF14	2.81	4.23
IFT74	2.80	2.83
RASA3	2.78	4.14
HAUS2	2.77	2.94
SKA1	2.76	3.89
RELL1	2.74	2.59
CCDC124	2.72	5.26
SIRPA;SIRPB	2.72	3.37
PVRL2	2.71	3.44
TBC1D10B	2.67	4.94
CD300LF	2.65	3.17
LARP4B	2.60	3.37
C2CD5	2.60	4.10
LTV1	2.60	5.26
MARK3	2.59	4.79
CLASP1	2.57	4.59
TNRC6B	2.51	3.05
HMHA1	2.49	3.66
AGTPBP1	2.46	4.16
MARK2	2.46	3.78
PIK3C2B	2.42	5.30
CNOT10	2.39	4.01
ABI1	2.37	4.03

Table S2. MYO1E BioID data. Gene IDs, difference over control and p-value scores are shown

Myo1E-control		
Gene name	Difference	-Log p value
MYO1E	11.04	6.72
ITGA2	7.42	4.98
ITGA5	7.40	6.70
FAM171A1	6.95	4.55
ITGAV	6.85	3.73
SNAP23	6.55	6.64
NOTCH2	6.42	6.03
PVRL3	6.30	7.80
STEAP3	6.28	5.29
RASAL2	6.02	5.41
ROBO1	5.93	7.74
SH3BP4	5.91	4.01
CD151	5.91	3.11
SLC30A1	5.71	3.19
VANGL1	5.71	4.69
ZDHHC5	5.68	6.92
HLA-A	5.62	5.00
VAMP5	5.60	2.99
PVRL2	5.57	5.66
ITGB5	5.48	3.41
DAG1	5.48	6.56
PPFIBP1	5.46	3.60
CDC42EP1	5.45	7.00
PHACTR4	5.33	4.98
SCRIB	5.32	3.69
DCBLD2	5.29	6.90
KIRREL	5.06	6.70
SLC7A5	5.04	2.50
SHB	5.03	6.71
SLC9A3R2	5.01	4.94
UACA	5.01	5.57
RASA3	4.97	4.82
FERMT2	4.91	5.28
RELL1	4.88	5.45
ATP2B4	4.84	5.40
SLC3A2	4.80	5.58
PARD3	4.80	6.45
PKN2	4.79	5.34
EFNB2	4.78	6.36
SLC4A7	4.76	5.44
RAB27B	4.75	5.42
MARK2	4.75	5.14
CSPG4	4.71	5.24
PAK4	4.67	3.00
JAG1	4.66	6.03
MARK3	4.60	3.92
VEPH1	4.54	6.13
ROCK1	4.52	4.82
ITGA3	4.45	7.84
EFR3A	4.43	4.90
LLGL1	4.42	7.59
GPR176	4.40	5.46
PCDH7	4.39	8.28
KIAA1522	4.38	5.17
CDCA3	4.37	5.29
PTPRJ	4.36	2.91
YES1	4.36	6.03
ANTXR2	4.35	4.88
ITGA6	4.35	4.86
USP6NL	4.34	6.13
CDC42BPA	4.32	4.27
SNX9	4.32	5.73
MPZL1	4.32	5.31
PACSIN2	4.26	6.05
ITGA7	4.26	6.10
RGL2	4.25	5.02
CASKIN2	4.25	5.90
ITGB1	4.24	8.68
PCDH10	4.22	5.09
DOCK10	4.19	4.15
CTNNA1	4.16	4.30
CTNND1	4.15	6.61
SIRPA;SIRPB	4.15	5.15
PTPN14	4.14	4.04
RALGAPA1	4.11	4.31
CDC42EP4	4.09	4.54
IL6ST	4.05	5.22
BSG	4.04	3.77
TENM3	4.01	5.93
RICTOR	4.01	5.55
MXRA8	3.98	5.44
TXNL1	3.98	3.70
SLC39A6	3.98	6.21
SLC12A2	3.96	6.39
CD44	3.96	6.27
NF2	3.96	4.28
AHCYL1	3.95	2.46
CPNE8	3.92	5.31
KCNMA1	3.92	5.41
SLC7A11	3.88	4.33
SPECC1	3.87	4.39
DIAPH3	3.86	3.15
EVI2B	3.86	4.51
PLSCR3	3.85	6.32

Myo1E ΔSH3-control		
Gene name	Difference	-Log p value
MYO1E	8.46	5.79
FAM171A1	7.59	4.80
VANGL1	6.59	6.01
RBMX;RBMX	6.39	6.83
ITGAV	6.38	4.00
ITGA2	5.49	4.13
PVRL3	5.37	6.87
SH3BP4	5.02	3.57
STEAP3	4.90	4.73
ITGB5	4.86	3.44
UACA	4.77	5.36
ACACB	4.69	5.50
DBT	4.68	4.92
TXNL1	4.60	4.03
ABCC1	4.57	4.71
RPL36	4.53	3.33
NOTCH2	4.51	4.96
CD151	4.48	2.47
SLC30A1	4.43	2.69
SLC4A7	4.43	5.22
ROBO1	4.39	6.44
SNAP23	4.26	5.19
PVRL2	4.06	4.72
PPFIBP1	4.02	2.74
KIRREL	3.99	5.99
ZDHHC5	3.97	5.64
ASPH	3.95	7.08
VAMP5	3.94	2.19
SCRIB	3.90	2.88
RPS5	3.85	6.50
CDC42EP1	3.83	5.97
RASAL2	3.83	3.78
ITGA5	3.79	3.81
EFNB2	3.78	5.43
PAK4	3.74	2.46
CSPG4	3.73	4.69
CANX	3.73	4.22
RELL1	3.72	4.70
RPL26;RPL26	3.70	1.74
RPL38	3.63	2.45
DCBLD2	3.59	5.79
SEMA7A	3.59	1.69
PTPRJ	3.50	2.36
YES1	3.49	4.66
EFR3A	3.46	4.24
RAC1;RAC3;F	3.43	3.99
BSG	3.42	4.02
FN1	3.41	4.13
SHB	3.39	5.25
GPR176	3.39	4.74
SLC7A5	3.39	1.68
TCEB2	3.37	1.94
PARD3	3.36	5.41
SNTB1	3.35	4.52
PALM2	3.23	4.42
FAM171B	3.19	4.54
DLG5	3.19	3.26
PHACTR4	3.16	3.47
SIRPA;SIRPB	3.14	4.25
ITGA3	3.11	6.95
SPECC1	3.09	4.02
FERMT2	3.06	3.95
RPL27	3.06	1.91
CDC42BPA	3.05	2.95
ATP2B4	3.05	4.06
RPS18	3.04	2.61
SLC3A2	3.02	4.26
RPL35	3.02	1.26
DOCK10	3.01	3.47
JAG1	3.01	4.63
RPLP1	2.99	1.03
NT5E	2.98	1.85
GNB2	2.96	1.96
RPL34	2.95	1.75
MARK3	2.94	2.54
HSPD1	2.93	2.26
RPS17	2.92	5.08
RPS13	2.88	1.64
MPZL1	2.88	4.15
ANTXR2	2.87	3.67
SLC9A3R2	2.86	3.77
RPS19	2.84	2.77
CTNND1	2.83	5.26
EPB41	2.82	6.24
VEPH1	2.82	5.05
DST	2.81	2.18
DSG2	2.80	7.32
ADAM9	2.78	3.74
ITGB1	2.76	7.58
DDOST	2.76	4.59
FLOT2	2.75	1.68
GNB1	2.74	1.57
PCDH10	2.73	4.16
CD44	2.73	4.96

Myo1E ΔPH-control		
Gene name	Difference	-Log p value
MYO1E	10.94	6.84
ITGAV	7.23	4.39
ITGA5	6.47	6.81
ITGA2	6.29	4.48
FAM171A1	6.27	4.22
ITGB5	5.91	4.03
SNAP23	5.84	6.39
SH3BP4	5.76	3.95
PVRL3	5.71	7.35
ACTBL2	5.59	3.58
RBMX;RBMX	5.56	6.23
CD151	5.51	2.98
NOTCH2	5.50	5.56
VANGL1	5.50	5.52
ROBO1	5.36	7.72
PVRL2	5.25	5.27
RASAL2	5.16	5.22
ZDHHC5	5.12	6.59
SLC30A1	5.12	3.05
BSG	4.98	5.10
STEAP3	4.94	4.14
VAMP5	4.87	2.66
PHACTR4	4.87	4.72
DCBLD2	4.85	6.52
CDC42EP1	4.82	6.49
HLA-A	4.80	4.55
PPFIBP1	4.71	3.22
SLC7A5	4.67	2.33
SNX33	4.64	4.43
CD99	4.59	6.58
RELL1	4.56	5.34
PKN2	4.54	5.26
FERMT2	4.50	5.00
DAG1	4.48	5.61
SCRIB	4.46	3.24
KIRREL	4.38	6.00
PAK4	4.36	2.82
EFNB2	4.30	5.95
PARD3	4.30	6.41
DIAPH3	4.27	3.50
GPR176	4.27	5.42
ATP2B4	4.25	5.02
RPS26;RPS26	4.25	2.18
SNX9	4.25	5.57
ITGA3	4.22	7.43
RGL2	4.18	4.93
SLC4A7	4.14	4.99
RASA3	4.13	4.22
SLC3A2	4.07	5.08
CDCA3	4.07	5.03
PTPN14	4.07	3.96
LLGL1	4.02	7.43
SIRPA;SIRPB	4.01	5.24
TMEM2	3.97	4.14
PTPRJ	3.97	2.64
ITGA7	3.96	5.55
VEPH1	3.95	6.01
SHB	3.94	5.07
EFR3A	3.90	4.55
RPL38	3.90	2.54
CSPG4	3.89	4.47
KIAA1522	3.87	5.04
SNX18	3.85	5.85
MARK2	3.82	4.48
SLC9A3R2	3.80	4.15
ITGA6	3.79	4.47
MPZL1	3.76	4.92
FKRP	3.75	1.85
ITGB1	3.75	7.79
IL6ST	3.74	5.07
YES1	3.69	5.46
CTNND1	3.69	6.23
UACA	3.69	4.65
NF2	3.68	4.11
USP6NL	3.67	5.51
CASK	3.66	4.18
TENM3	3.65	5.76
PACSIN2	3.65	5.58
EHD2	3.62	2.99
HLA-C	3.56	3.28
PLSCR3	3.54	5.83
CTNNA1	3.50	3.92
AHCYL1	3.46	2.16
ASPH	3.46	4.99
JAG1	3.45	5.16
PALM2	3.44	4.47
SLC39A6	3.43	6.30
ADAM9	3.41	4.34
C2CD2	3.39	4.53
SLC39A10	3.39	4.60
SNTB1	3.39	4.93
EPS8	3.38	4.70
MARK3	3.38	3.14
CDC42EP4	3.35	3.92

SNTB1	3.84	4.26	C2CD2	2.73	3.90	RPS5	3.34	3.90
FMNL3	3.83	4.15	KCNMA1	2.72	4.33	DOCK10	3.33	3.85
SLC39A10	3.81	4.83	CASK	2.65	3.28	CASKIN2	3.33	5.44
HLA-C	3.80	3.46	RPL36AL	2.65	0.97	SPECC1	3.32	4.25
ADAM9	3.77	4.63	ILF3	2.65	4.33	KCNMA1	3.29	4.93
ANKRD50	3.76	6.67	EHD2	2.63	2.23	GAB1	3.28	5.28
SNX18	3.69	5.79	DAG1	2.62	2.70	CTNNB1	3.27	4.47
SNX33	3.66	3.75	FMNL3	2.62	3.66	S100A6	3.25	0.83
PRR16	3.64	5.10	RPS25	2.60	1.97	OCC1	3.23	4.54
EHD2	3.61	2.93	SLC39A10	2.59	3.82	ATP2B1	3.23	3.31
FGD6	3.59	4.89	CDC42	2.58	2.58	EVI2B	3.22	4.00
GAB1	3.54	5.51	FCHO2	2.57	4.78	CXADR	3.22	3.79
TMEM2	3.54	3.70	NF2	2.55	3.05	CD44	3.22	5.51
ZDHHC20	3.53	5.55	JUP	2.52	4.31	ROCK1	3.21	3.91
ERBB2	3.53	5.96	H2AFY	2.51	1.98	FRMD6	3.20	2.98
C2CD2	3.52	4.61	RPS10;RPS1(	2.51	2.46	ANTXR2	3.20	4.05
EGFR	3.52	6.84	EPHA2	2.51	5.75	MXRA8	3.19	4.83
SLITRK5	3.49	3.64	SLC39A6	2.51	2.56	FGD6	3.18	4.55
MTMR1	3.49	4.25	RPL32	2.48	1.70	PRR16	3.18	4.90
PALM2	3.47	4.39	AKAP12	2.45	2.06	RICTOR	3.14	4.82
FCHO2	3.45	5.72	PLSCR3	2.44	4.99	PCDH7	3.13	7.23
VASN	3.43	5.20	RRAS2	2.43	1.57	KCNN4	3.12	5.50
ATP2B1	3.43	3.50	HLA-A	2.43	1.09	ZDHHC20	3.11	4.83
GPRIN1	3.43	2.46	FLOT1	2.43	1.72	SDC1	3.11	2.36
RAB23	3.42	5.15	RPS26;RPS2(	2.42	0.75	RASA2	3.11	3.81
GULP1	3.41	2.63	CACNA2D1	2.42	1.37	MTMR1	3.09	3.88
ROR1	3.40	6.40	PTPN14	2.41	2.57	SLC16A3	3.06	5.04
SLC16A3	3.37	5.48	GNG12	2.41	1.38	EGFR	3.04	6.42
LPHN2	3.37	3.60	UTRN	2.41	2.11	FMNL3	3.02	3.91
JUP	3.36	5.21	ACACA	2.41	6.51	ATP1B3	3.02	3.96
MAP4K5	3.34	5.74	SLC12A2	2.39	4.58	CDC42BPA	3.01	3.35
CTNNB1	3.32	4.14	PKN2	2.37	3.46	FCHO2	2.98	5.27
WIPF2	3.32	4.48	PTRF	2.36	1.61	SLC20A2	2.96	4.82
WDR20	3.29	5.15	SLC16A3	2.35	4.47	SLC12A2	2.94	5.50
SLC20A1	3.29	3.81	SNX9	2.32	3.93	CDC42	2.94	2.70
PLXNA1	3.29	4.28	FGD6	2.32	3.67	MAP4K3	2.93	5.02
ESYT2	3.28	3.02	HIST1H1B	2.31	1.06	WIPF2	2.93	4.04
SLC39A14	3.28	5.29	MCCC2	2.31	3.01	ECE1	2.91	4.10
FLOT1	3.28	2.31	LGALS1	2.30	1.15	SLC7A11	2.91	3.71
ARHGAP32	3.25	4.26	CAV1	2.30	1.03	MAP4K5	2.90	5.24
SBF1	3.23	5.61	RPL30	2.29	4.30	CDC42EP3	2.89	5.67
USP12	3.22	4.57	KANK1	2.28	1.54	AMIGO2	2.89	5.42
RASA2	3.20	3.75	ITGA6	2.27	2.89	ROR1	2.89	6.20
KIAA1549L	3.20	5.11	EGFR	2.26	5.53	ABI2	2.88	1.94
ABCC1	3.18	3.69	PCCA	2.25	6.30	PCDH10	2.88	4.30
SLC1A5	3.17	4.26	RPL18	2.25	0.95	WASL	2.86	2.14
KIDINS220	3.16	3.70	RPLP2	2.23	1.17	LZTS2	2.85	5.19
DSG2	3.15	7.27	KIDINS220	2.21	3.19	SLC1A5	2.81	3.90
CXADR	3.15	4.07	SNTB2	2.21	2.02	ZDHHC8	2.79	4.22
CDH2	3.07	5.73	PLAUR	2.20	1.36	ARF6	2.79	1.65
YKT6	3.06	5.42	HYOU1	2.19	3.45	USP12	2.76	4.09
PEAK1	3.05	2.33	MARK2	2.18	2.29	MTMR10	2.75	3.89
STIM2	3.04	4.62	ATP2B1	2.16	2.36	WDR20	2.73	4.99
RHBDF1	3.04	4.34	LLGL1	2.15	3.44	MAPKAP1	2.72	4.33
PLCB3	3.03	4.52	ARHGAP32	2.13	3.11	FLOT1	2.71	1.93
PTPN13	3.03	3.38	MXRA8	2.13	3.67	SLC16A1	2.70	1.62
CASK	3.03	2.49	PCCB	2.13	5.36	ARHGAP22	2.65	4.16
SLC4A8	3.02	3.64	NCEH1	2.11	3.18	MT-CO2	2.64	2.81
DBT	3.02	2.90	HNRNPC	2.10	1.59	JUP	2.63	4.32
EPB41L5	3.00	6.23	ITGA7	2.10	3.49	RAB23	2.63	4.44
EPS8	3.00	4.36	MCCC1	2.09	7.55	SBF1	2.61	5.00
ZDHHC8	2.99	4.28	ROCK1	2.08	2.63	MET	2.61	4.40
FRMD6	2.99	2.87	ERBB2IP	2.08	5.20	DLG1	2.61	3.89
CD99	2.98	1.52	SDC1	2.08	0.82	BCAR3	2.60	2.22
DLG1	2.96	4.34	SLC1A5	2.08	3.07	MAGI1	2.59	3.23
MET	2.96	4.68	TRIOBP	2.07	3.96	RPS18	2.58	2.05
UTRN	2.94	2.46	DLG1	2.05	3.31	DHX30	2.56	4.75
FAT4	2.93	3.69	GNAI2	2.04	1.89	DLC1	2.55	3.03
DLG5	2.92	2.91	MYO1B	2.03	2.63	TNS1	2.55	2.78
VAMP3	2.91	5.02	RPL10A	2.02	4.60	ESYT2	2.55	2.40
PPFIA1	2.90	3.22	SLC4A8	2.01	1.84	CPNE8	2.55	3.95
ARHGAP22	2.90	4.73	CYB5R3	2.01	1.00	KIAA1549L	2.54	4.83
CLMP	2.89	5.00	ALCAM	2.00	1.43	TENM4	2.52	2.44
AKAP12	2.88	2.29	HIST1H2AJ;H	2.00	2.39	PHACTR2	2.52	5.12
WASL	2.88	2.19	MINK1	2.00	3.10	DSG2	2.49	6.12
MINK1	2.86	4.34	RPL6	1.99	5.31	EPB41L5	2.49	5.68
BAIAP2	2.83	5.34	IQGAP1	1.98	2.62	RAB27B	2.48	1.56
DOS	2.81	6.06	RASA3	1.97	1.79	PEAK1	2.48	1.92
EPHA2	2.81	5.96	PEAK1	1.97	1.47	LPHN2	2.48	2.84
SLC20A2	2.79	4.87	SLITRK5	1.96	2.30	CNP	2.47	2.73
PAG1	2.78	4.06	CD59	1.95	1.87	VASN	2.46	4.35
SNTB2	2.77	2.52	RPN1	1.95	1.52	VAT1	2.45	4.19
VAT1	2.77	4.36	APBB2	1.95	1.65	VAMP3	2.43	4.62
ITGA1	2.72	6.52	PHB	1.94	1.12	RAP1B;RAP1	2.43	1.22
RPL26;RPL26	2.72	1.26	EPB41L2	1.93	5.25	BAIAP2	2.43	4.84
CDC42EP3	2.70	5.76	RICTOR	1.89	2.68	FAM171B	2.43	3.64
PHACTR2	2.68	4.66	ATP1A1	1.89	1.33	YKT6	2.43	4.10
MAGI1	2.68	3.35	SBF1	1.89	3.27	GULP1	2.42	1.95
ARHGAP21	2.67	1.80	ACSL4	1.87	1.42	ALCAM	2.42	3.14
LZTS2	2.66	4.21	DLST	1.86	3.22	PLCB3	2.41	3.94
SLC16A1	2.65	1.56	RAB34	1.84	2.09	RAB27A	2.40	1.95
CDC42BPB	2.64	3.00	IPO5	1.81	1.50	C2CD5	2.40	3.62
NF1	2.64	2.60	CXADR	1.80	2.82	HSD17B12	2.39	1.89
KIAA0754	2.61	3.03	HSP90B1	1.79	1.70	CANX	2.38	1.64
SLC9A3R1	2.60	2.01	RAB11B;RAB	1.78	1.33	ROCK2	2.38	3.01
ARL13B	2.59	6.17	FAM171A2	1.77	1.65	EPHA2	2.35	5.27
KCNN4	2.58	5.08	RPL7A	1.76	2.28	PPFIA1	2.34	2.75

Journal of Cell Science • Supplementary information

CCDC88A	2.56	1.72
ERBB2IP	2.56	5.55
ABI2	2.55	1.68
PLEKHA5	2.54	4.40
CLDND1	2.52	1.32
EPB41L1	2.47	2.86
CEP89	2.45	4.43
FAM129B	2.40	4.91
FLOT2	2.39	1.19
TBC1D8	2.38	1.83
TRIOBP	2.38	3.69
PTRF	2.37	1.61
BASP1	2.35	4.43
DOCK9	2.35	3.74
C1orf21	2.34	3.32
C2CD5	2.34	3.53
PLCB1	2.34	4.40
CAV1	2.32	1.04
PSD3	2.31	3.17
DEPDC1B	2.31	4.51
MTMR10	2.31	2.39
RAB11B;RAB	2.27	1.74
NOTCH1	2.26	1.73
TBC1D10B	2.25	2.64
BCAR3	2.24	1.78
TNFRSF11A	2.24	2.91
OCC1	2.23	1.45
RAI14	2.22	4.59
EPB41	2.20	5.46
CYFIP2	2.20	1.37
TULP3	2.19	2.22
PI4KA	2.19	3.38
EPB41L2	2.19	5.59
MAP4K3	2.18	1.42
DLC1	2.17	2.52
ROCK2	2.17	2.02
MARCKS	2.16	5.42
NUMB	2.16	6.92
EPHA4	2.15	2.76
EHD1	2.13	4.27
RAB34	2.10	2.39
FIBP	2.08	2.92
ANKS1A	2.05	1.66
ZC3HAV1	2.01	3.24
KIAA1217	2.00	1.59
MCCC2	1.99	2.28
RRAS2	1.96	1.20
ADD3	1.95	4.32
MAP4K4	1.94	3.49
ATP1A1	1.93	1.35
DOCK5	1.92	3.15
APBB2	1.91	1.61
SPRED2	1.89	2.75
SLC29A1	1.87	1.22
FAM171B	1.86	1.52
ABI1	1.86	3.15
KIAA1549	1.85	1.49
MYOF	1.82	3.52
RPS27A;UBA	1.82	4.59
ADD1	1.81	4.51
RASSF8	1.81	1.18
ARHGAP39	1.81	5.47
FNBP1	1.80	1.63
SNAP29	1.79	5.46
ASAP1	1.75	4.53
TP53BP2	1.74	1.37
STXBP1	1.74	3.08
C2CD2L	1.74	2.56
SRGAP1	1.73	1.43
AMIGO2	1.70	1.40
LIN7C	1.68	1.18
SIPA1L3	1.68	1.36
EPS15	1.68	1.99
CDK5	1.67	3.17
MPP5	1.65	2.65
CYFIP1	1.64	3.99
CDC42	1.64	1.22
COBLL1	1.62	1.22
WASF2	1.62	3.98
DLGAP4	1.61	1.45
NCKAP1	1.59	5.03
DAB2	1.56	5.38

ESYT2	1.75	1.65
FRMD6	1.74	1.68
RPLP0;RPLP0	1.73	3.65
RPL8	1.72	4.60
PTPN13	1.72	2.25
RPL12	1.72	5.31
P4HA1	1.71	1.64
RAI14	1.67	7.29
RPL35A	1.65	4.05
PC	1.64	4.17
VAMP3	1.58	3.41

ANKRD50	2.30	3.68
RAB10	2.30	1.51
VDAC3	2.28	1.05
RAB34	2.28	2.55
SLC25A4	2.26	1.69
PDGFRB	2.26	3.84
RPS13	2.26	1.13
RPL36	2.25	1.20
DHCR24	2.25	3.43
ATP1A1	2.24	1.62
RAB11B;RAB	2.24	1.63
TXNIP	2.22	2.88
RPN1	2.22	1.70
ERBB2IP	2.22	5.46
PTRF	2.21	1.50
TMEM165	2.20	1.49
DOCK5	2.19	3.51
DOCK9	2.18	3.61
BASP1	2.18	4.17
ITSN2	2.18	2.30
TLDC1	2.17	2.71
YWHAG	2.17	1.45
ARHGAP32	2.16	3.60
SAR1A	2.16	1.27
FIBP	2.16	2.92
LNPEP	2.15	3.39
DLG5	2.14	2.31
TM9SF3	2.13	1.36
EHD4	2.10	5.31
SLC4A8	2.10	2.69
RPS25	2.07	1.40
TBC1D10B	2.07	2.57
CYFIP2	2.06	1.29
SNTB2	2.06	1.87
UTRN	2.06	1.79
SLC20A1	2.05	1.35
RPN2	2.04	1.57
PTPN13	2.04	2.52
SLC9A3R1	2.03	1.51
ABI1	2.03	3.83
CEP89	2.02	4.62
APBB2	2.01	1.71
HSPD1	1.98	1.40
ERBB2	1.97	4.89
XPO1	1.95	1.97
DNAJA1	1.93	2.77
KIAA0754	1.91	2.57
PLEKHA5	1.90	3.84
EPB41L2	1.88	5.07
MARCKS	1.86	5.47
ANKS1A	1.85	1.51
CDH2	1.84	1.55
SLC12A4	1.84	2.80
FRS2	1.83	3.25
NCEH1	1.83	1.61
FAM129B	1.82	4.36
AKAP12	1.81	1.63
ASAP1	1.81	4.62
TRAM1	1.80	1.81
UQCRC2	1.78	2.01
DNAJA2	1.77	3.69
DLGAP4	1.76	1.75
STIM2	1.76	1.78
ARL3	1.74	2.17
EHD1	1.73	3.75
PI4KA	1.73	2.91
CYFIP1	1.67	5.12
ARHGAP39	1.66	5.75
WASF2	1.65	5.08
NUMB	1.65	6.35
NCKAP1	1.59	6.18

Journal of Cell Science • Supplementary information

Table S3. List of proteins shown in the Venn diagram of Fig. 2E

MYO1F U937	MYO1F RPE	MYO1E RPE	MYO1F U937/ MYO1F RPE	MYO1E RPE/ MYO1F RPE	MYO1E RPE/MYO1F RPE MYO1F U937
SNAP29	WASL	PTPN14	CD44	FERMT2	PHACTR4
DLG1	JUP	CDC42EP4	ASAP1	ITGA7	MARK2
GOLGA8R	SLC39A10	DOCK10	MYO1F	PVRL3	ZDHHC5
MAP4K4	FRMD6	RAB27B	NF2	ROCK1	SLC4A7
PPFIBP2	ANKRD50	PACSIN2	CD99	ITGA5	SLC30A1
KANK2	BRK1	CDC42BPA		CD151	RASA3
ESYT1	PPFIA1	RICTOR		CSPG4	CDCA3
ADD3	KCNN4	SLC9A3R2		PCDH7	MARK3
ERBB2IP	CEP89	VEPH1		TENM3	SLC7A5
PECAM1	MAP4K5	IL6ST		SH3BP4	ITGA6
FYB	CDC42BPB	KIAA1522		ITGA2	
SH3KBP1	ADAM9	YES1		CDC42EP1	
MARCKS	SHISA2	USP6NL		CASKIN2	
GPR124	EHD2	MPZL1		EFNB2	
BASP1	ATP2B1	RALGAPA1		RASAL2	
SH3GL1	SLC12A2	BSG		RELL1	
SH3BP2	EGFR	LLGL1		ITGB1	
UTRN	VASN	SNX9		VAMP5	
CD2AP	SLITRK5			PARD3	
SIGLEC6				DAG1	
NUMB				PAK4	
AHCYL1				PCDH10	
ESYT2				CTNND1	
FAM129B				ITGAV	
CDC37				VANGL1	
FNBP1				ITGA3	
DOCK8				SHB	
WAS				HLA-A	
LAT2				SLC3A2	
PLEKHO2				CTNNA1	
				KIRREL	
				RGL2	
				PTPRJ	
				JAG1	
				ROBO1	
				ITGB5	
				ANTXR2	
				GPR176	
				SCRIB	
				ATP2B4	
				PVRL2	
				PPFIBP1	
				DCBLD2	
				SNAP23	
				UACA	
				FAM171A1	
				NOTCH2	
				MYO1E	
				PKN2	

Journal of Cell Science • Supplementary information

Figure S1 A raw blots

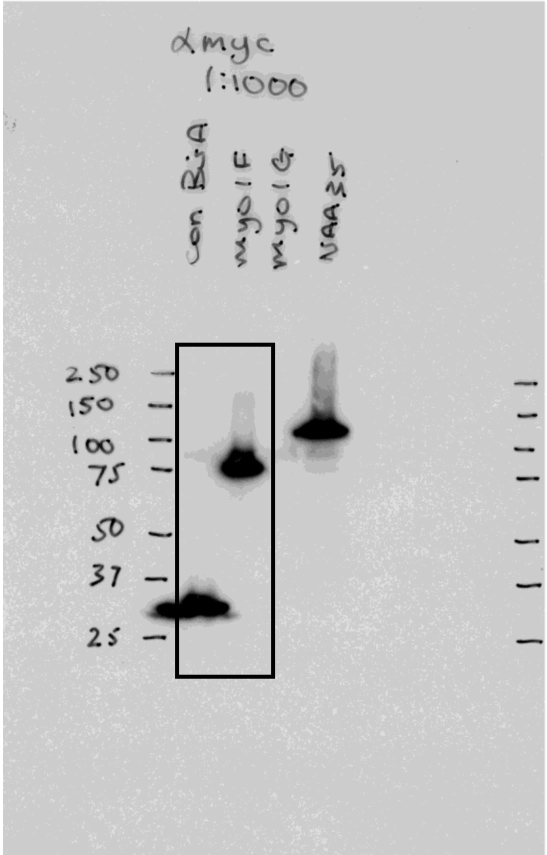

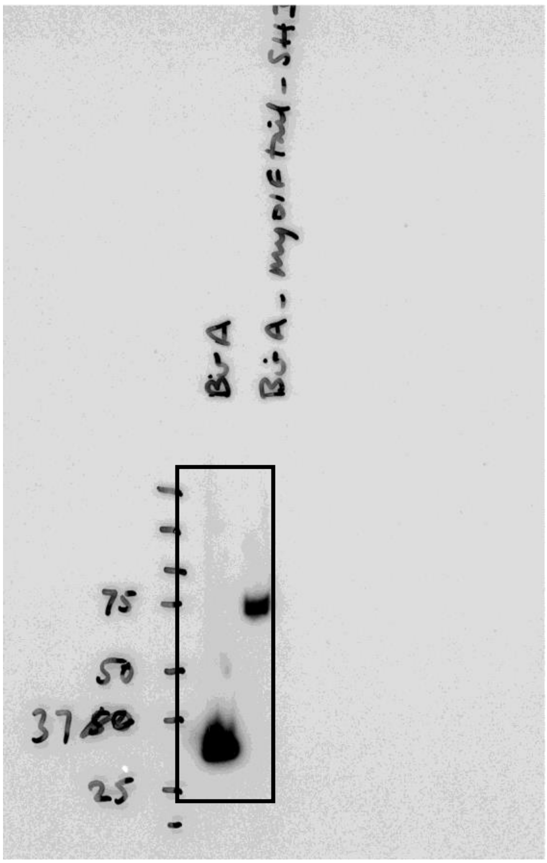

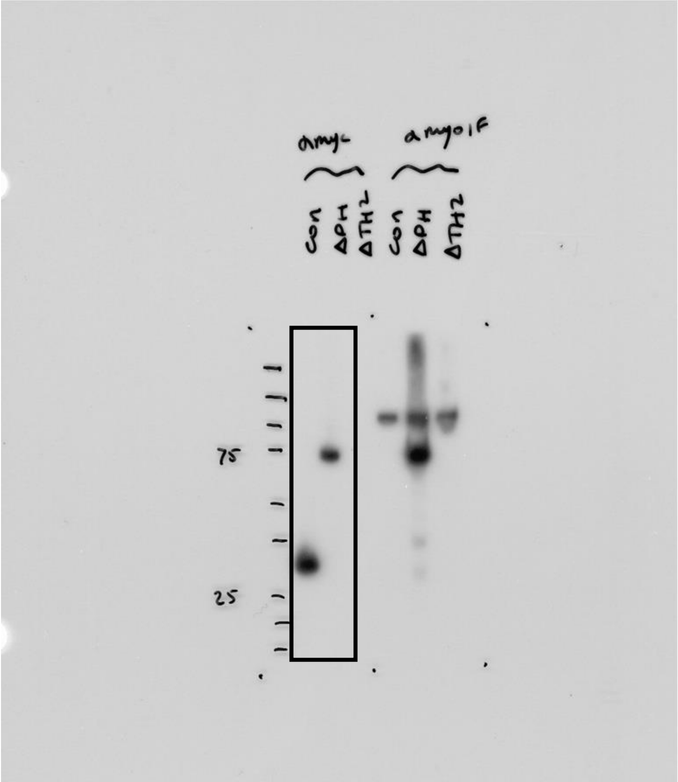

Journal of Cell Science • Supplementary information

Figure S1 B raw blots

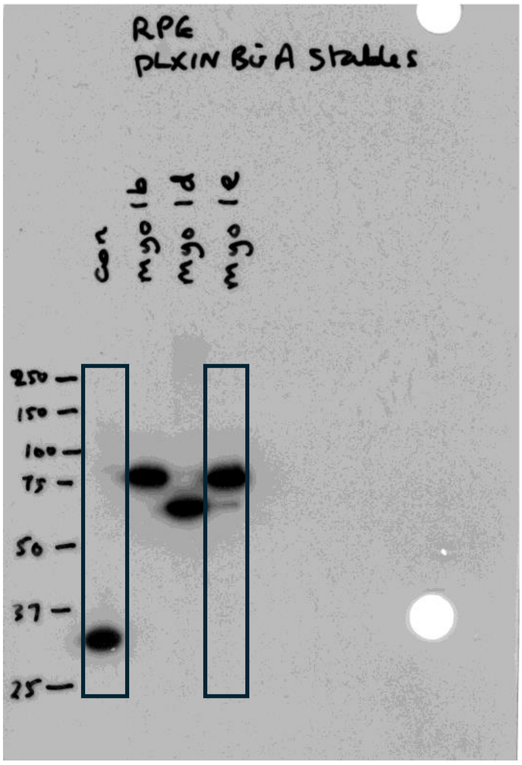

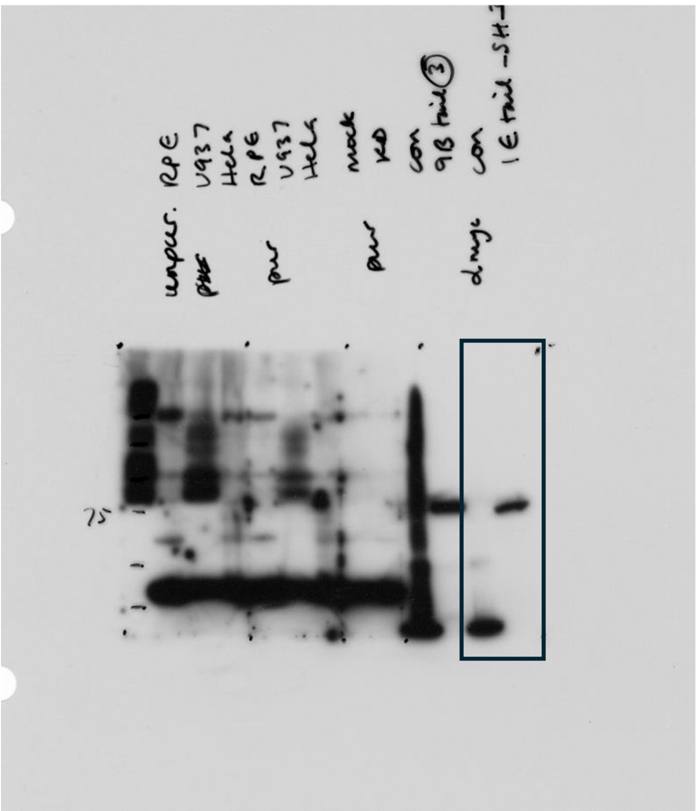

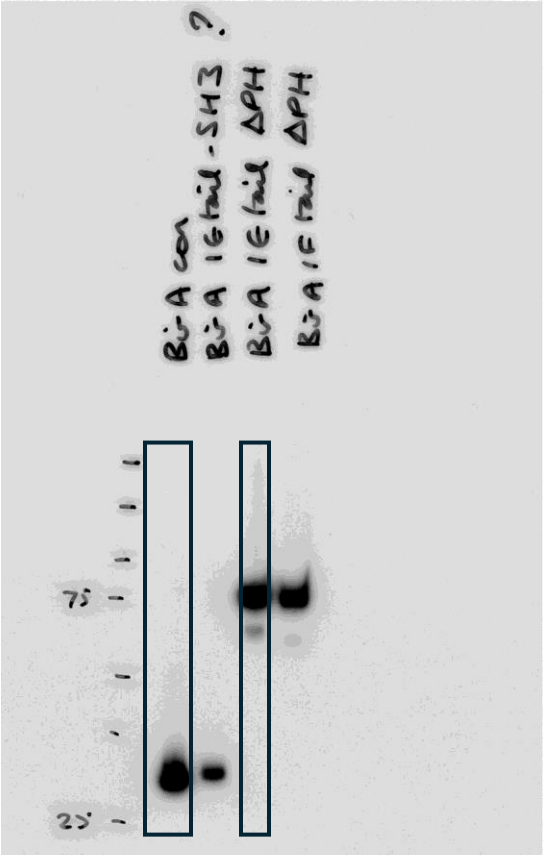

Fig 1A raw data

MYO1F

actin

IBA1

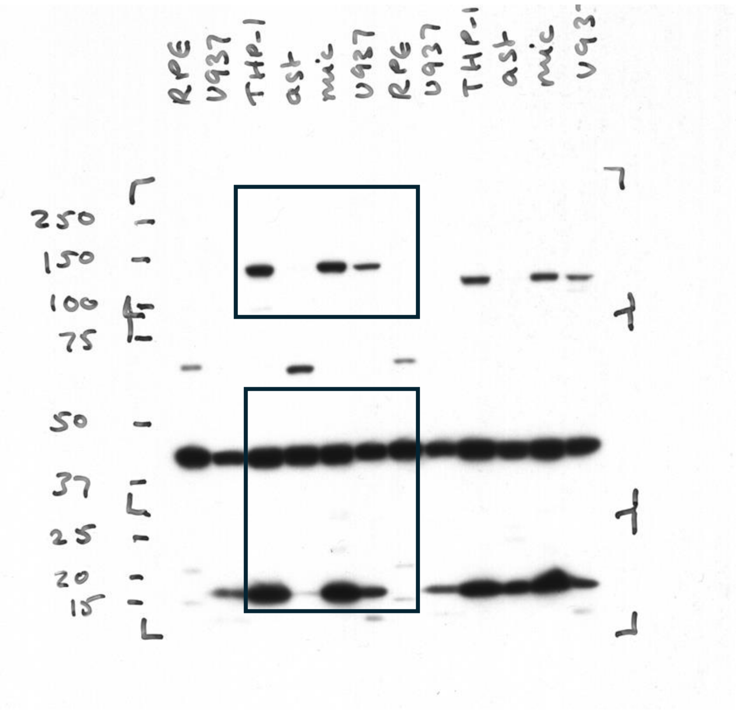

MYO1E

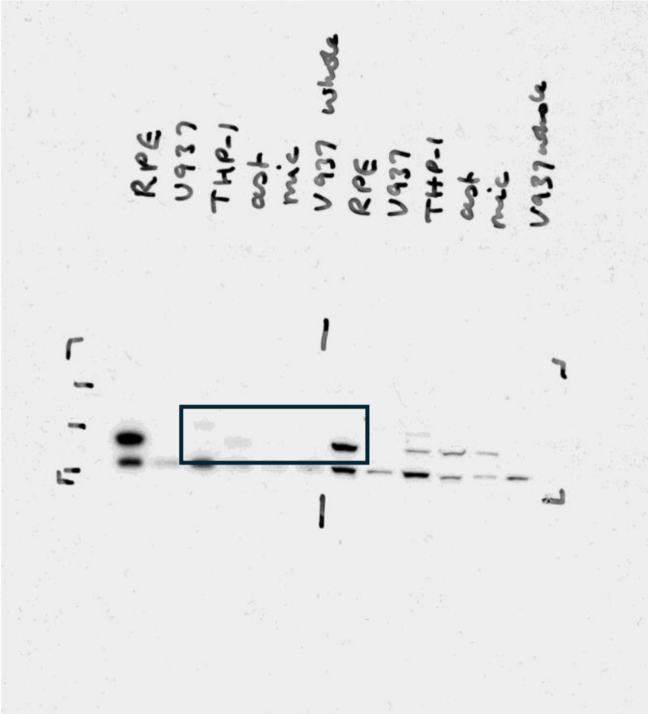

Fig 3C raw data

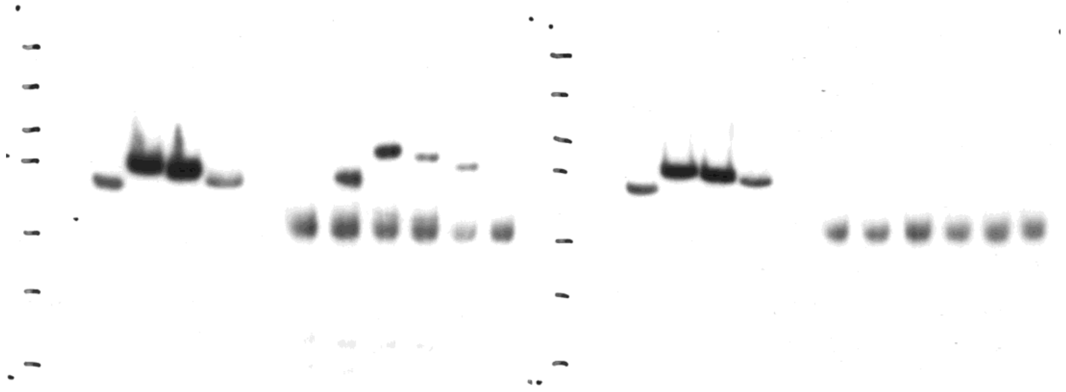

Journal of Cell Science • Supplementary information

Fig 3D raw data

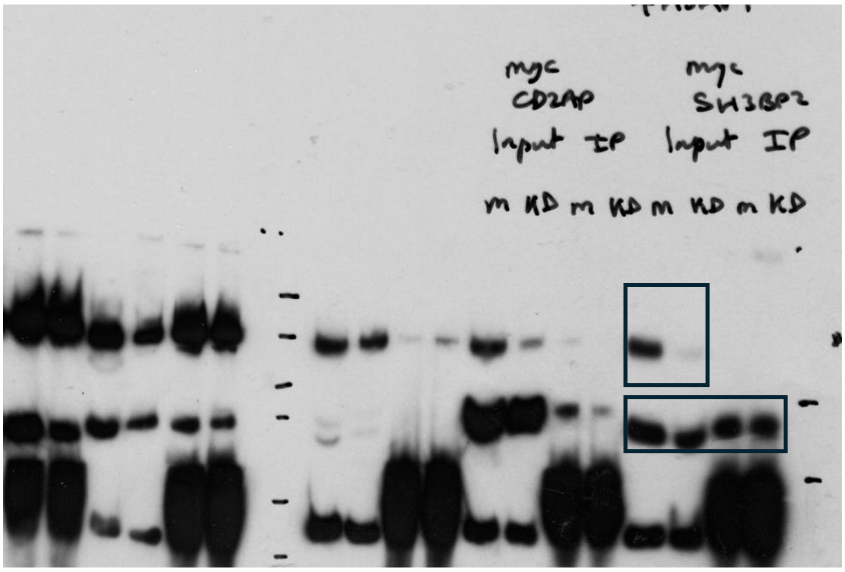

SH3KBP1

myc SH3BP2

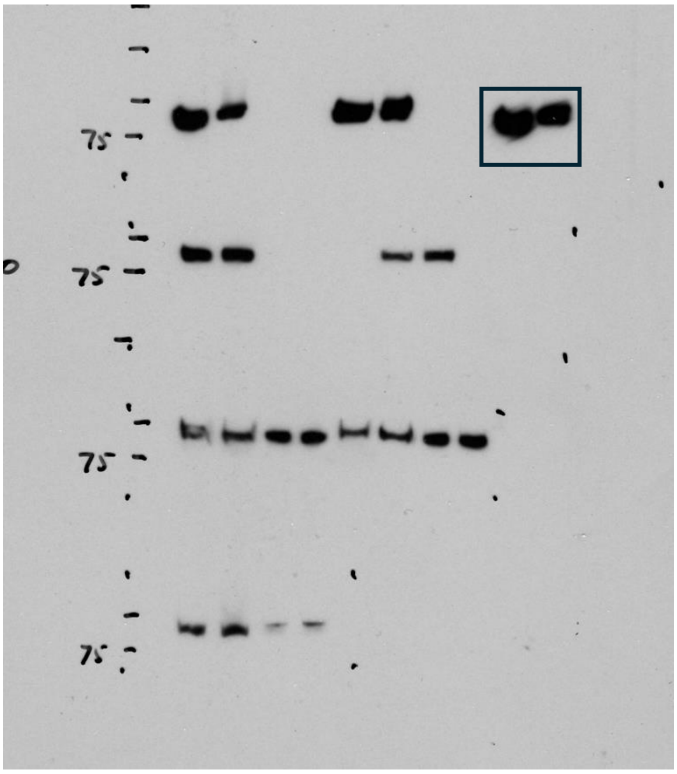

EF-2

Journal of Cell Science • Supplementary information

J. Cell Sci.: doi:10.1242/jcs.264357: Supplementary information

Fig 3E raw data

myc CD2AP

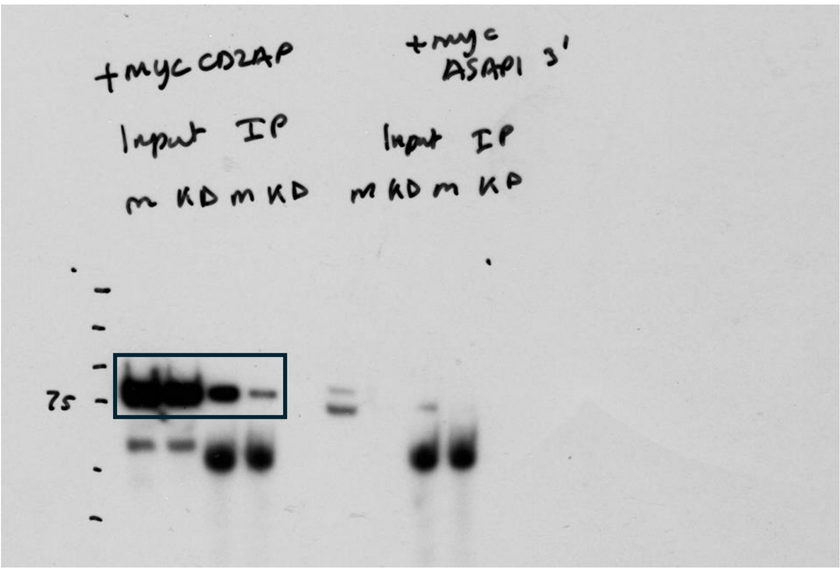

SH3KBP1

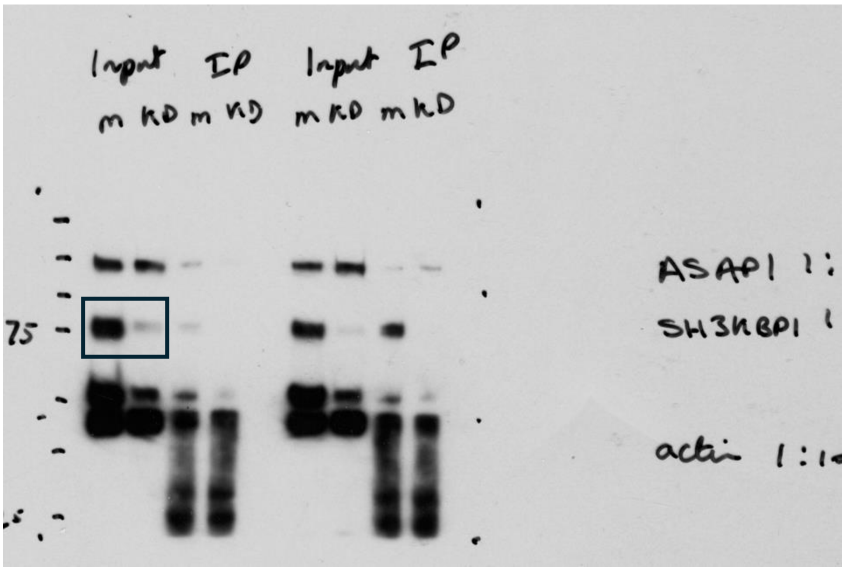

EF-2

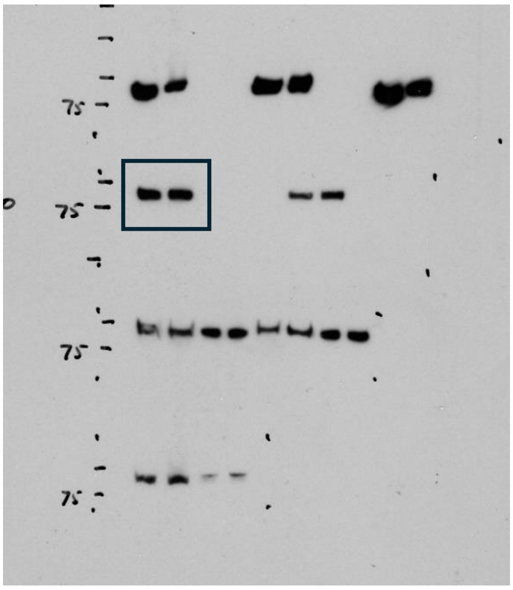

Journal of Cell Science • Supplementary information

Fig 3F raw data

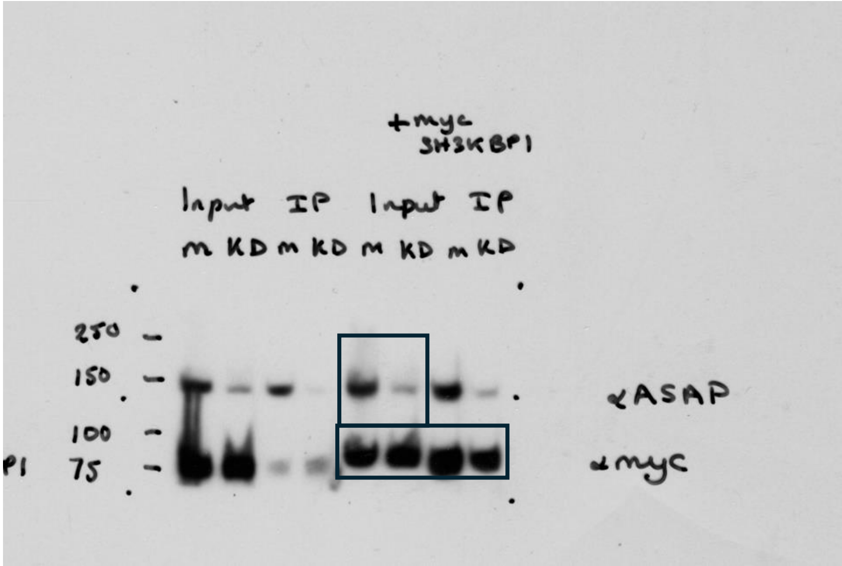

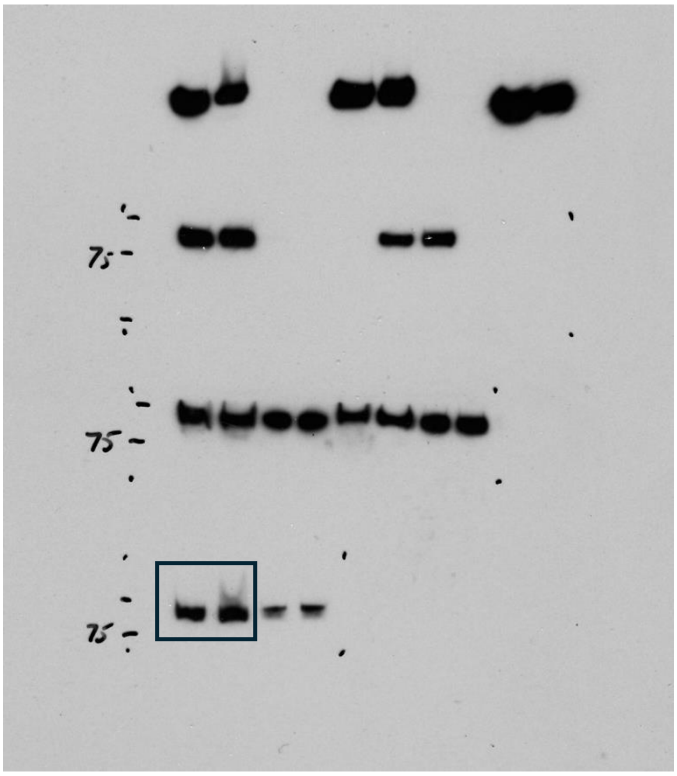

EF-2

Journal of Cell Science • Supplementary information

Fig 3G raw data

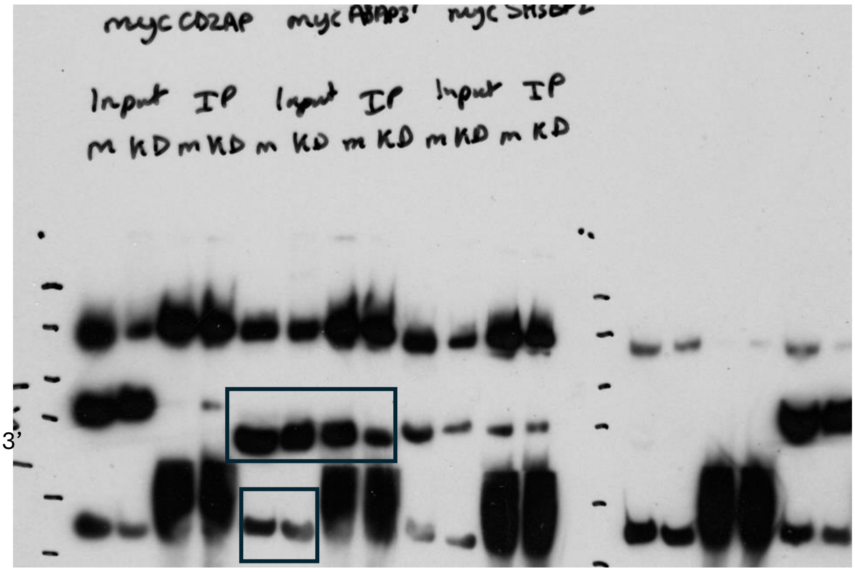

myc-ASAP 3'

actin

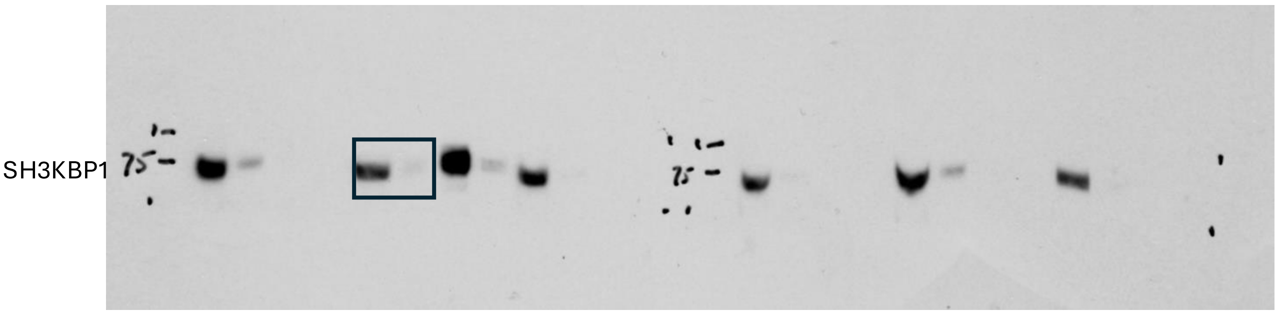

SH3KBP1

Journal of Cell Science • Supplementary information

Fig 5C raw data

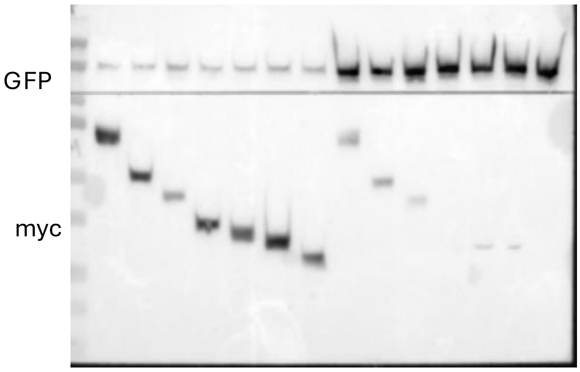

Fig. S9. Blot transparency

Journal of Cell Science • Supplementary information
